# Client and provider preferences for HIV care: Implications for implementing differentiated service delivery in Thailand

**DOI:** 10.1002/jia2.25693

**Published:** 2021-03-31

**Authors:** Sita Lujintanon, Sorawit Amatavete, Thanthip Sungsing, Pich Seekaew, Jitsupa Peelay, Pravit Mingkwanrungruang, Tanat Chinbunchorn, Somsong Teeratakulpisarn, Pornpen Methajittiphan, Prattana Leenasirima, Amarin Norchaiwong, Ampaipith Nilmanat, Praphan Phanuphak, Reshmie A Ramautarsing, Nittaya Phanuphak

**Affiliations:** ^1^ Institute of HIV Research and Innovation Bangkok Thailand; ^2^ Department of Epidemiology Columbia University Mailman School of Public Health New York NY USA; ^3^ Thai Red Cross AIDS Research Centre Bangkok Thailand; ^4^ Queen Savang Vadhana Memorial Hospital Chonburi Thailand; ^5^ Nakornping Hospital Chiang Mai Thailand; ^6^ Sarapee Hospital Chiang Mai Thailand; ^7^ Hatyai Hospital Songkhla Thailand; ^8^ Center of Excellence in Transgender Health Chulalongkorn University Bangkok Thailand

**Keywords:** differentiated service delivery, multi‐month dispensing, antiretroviral therapy, people living with HIV, Thailand

## Abstract

**Introduction:**

Differentiated service delivery (DSD) for antiretroviral therapy (ART) maintenance embodies the client‐centred approach to tailor services to support people living with HIV in adhering to treatment and achieving viral suppression. We aimed to assess the preferences for HIV care and attitudes towards DSD for ART maintenance among ART clients and providers at healthcare facilities in Thailand.

**Methods:**

A cross‐sectional study using self‐administered questionnaires was conducted in September‐November 2018 at five healthcare facilities in four high HIV burden provinces in Thailand. Eligible participants who were ART clients aged ≥18 years and ART providers were recruited by consecutive sampling. Descriptive statistics were used to summarize demographic characteristics, preferences for HIV services and expectations and concerns towards DSD for ART maintenance.

**Results:**

Five hundred clients and 52 providers completed the questionnaires. Their median ages (interquartile range; IQR) were 38.6 (29.8 to 45.5) and 37.3 (27.3 to 45.1); 48.5% and 78.9% were females, 16.8% and 1.9% were men who have sex with men, and 2.4% and 7.7% were transgender women, respectively. Most clients and providers agreed that ART maintenance tasks, including ART refill, viral load testing, HIV/sexually transmitted infection monitoring, and psychosocial support should be provided at ART clinics (85.2% to 90.8% vs. 76.9% to 84.6%), by physicians (77.0% to 94.6% vs. 71.2% to 100.0%), every three months (26.7% to 40.8% vs. 17.3% to 55.8%) or six months (33.0% to 56.7% vs. 28.9% to 80.8%). Clients agreed that DSD would encourage their autonomy (84.9%) and empower responsibility for their health (87.7%). Some clients and providers disagreed that DSD would lead to poor ART retention (54.0% vs. 40.4%), increased loss to follow‐up (52.5% vs. 42.3%), and delayed detection of treatment failure (48.3% vs. 44.2%), whereas 31.4% to 50.0% of providers were unsure about these expectations and concerns.

**Conclusions:**

Physician‐led, facility‐based clinical consultation visit spacing in combination with multi‐month ART refill was identified as one promising DSD model in Thailand. However, low preference for decentralization and task shifting may prove challenging to implement other models, especially since many providers were unsure about DSD benefits. This calls for local implementation studies to prove feasibility and governmental and social support to legitimize and normalize DSD in order to gain acceptance among clients and providers.

## 1 INTRODUCTION

Retention in care is critical to achieving the Joint United Nations Programme on HIV/AIDS (UNAIDS)’s 95‐95‐95 targets: 95% of those who are HIV positive are diagnosed, 95% of those diagnosed receive antiretroviral therapy (ART), and 95% of those treated achieve viral suppression by the end of 2030 [1]. To achieve these goals, nations have adapted their strategy to target vulnerable populations to link them to testing and treatment, and retain them in care. In Thailand, with an estimated 470,000 people living with HIV (PLHIV), only 80% of all PLHIV were on ART and 78% of PLHIV were virally suppressed in 2019 [2]. Consistent with global guidance, Thailand’s current practice to start ART for all PLHIV regardless of CD4 count [3] and pilot implementation of same‐day ART initiation [4] contribute to the growing cohort of PLHIV on ART. Studies show that the growing number of ART clients lays a burden on the ART recipients, such as experiencing a long waiting time for ART pick‐up, even for clinically stable clients, which may have a psychological and socioeconomic impact on their wellbeing and health outcomes [5, 6]. The growing number of clients also increases the healthcare provider workload and overwhelms the limited hospital capacity, which may consequently lead to lower quality patient‐provider interactions and compromise the health of PLHIV [7]. New approaches to supply ART to PLHIV are needed to reduce the burden on the healthcare system while maintaining the quality of care and to prevent poor retention in care due to suboptimal ART maintenance services.

The World Health Organization (WHO) recommends differentiated service delivery (DSD) for ART maintenance, which is a client‐centred approach to HIV service provision that adapts the services to meet the needs of ART recipients with diverse characteristics, clinical conditions, and preferences [8]. The DSD framework is characterized by four components: the type, the location, the provider, and the frequency of services, which are combined to create various DSD models that place a strong emphasis on decentralization, task shifting, and reducing visit frequency [9]. Most examples of differentiated maintenance services were documented in countries in sub‐Saharan Africa and are categorized into four models: client‐managed groups (i.e. community adherence groups), healthcare worker‐managed groups (i.e. adherence clubs), facility‐based individual models, which include appointment spacing and fast‐track drug refill, and out‐of‐facility individual models, which include community drug distribution points and home delivery [10, 11], with generally high retention and viral suppression of over 80% and 90% respectively [12]. A couple of examples were identified in Asia: telemedicine with e‐consultation and ART delivery via courier service has been available for stable clients at SHIP Clinic in the Philippines [13], and gay‐led, fee‐based ART maintenance service at G‐link Clinic in Vietnam has provided online counselling in addition to its facility‐based service [14]. In Thailand where men who have sex with men (MSM) and transgender women (TGW) are at heightened risk of HIV acquisition [15], decentralization and task shifting to key population (KP)‐led, community‐based organizations (CBOs) has been piloted and demonstrated to successfully reach HIV‐positive MSM and TGW, link them to care and maintain stable PLHIV at the CBOs [16]. However, other differentiated ART maintenance models, such as ART dispensing at the primary care centres and 6‐month clinical consultation as recommended in the 2017 Thailand National Guidelines on HIV/AIDS Treatment and Prevention [3], are not well documented. Little is known whether they are well received by Thai PLHIV. Since DSD emphasizes client centredness, it is crucial to understand what ART clients want in order to create differentiated models that are acceptable and meet their needs. Additionally, it is imperative to understand the preferences of Thai providers as their attitudes are critical when it comes to providing HIV services, its improvement, and further differentiation of service models.

This manuscript aims to assess the preferences for and attitudes towards DSD for ART maintenance among ART clients and providers at hospitals in Thailand.

## 2 METHODS

### 2.1 Study design and participants

In September‐November 2018, a cross‐sectional study using self‐administered questionnaires was conducted to assess the preferences in HIV care and attitudes towards DSD for ART maintenance. Client participants were ≥18 years, HIV‐positive, and receiving ART, and provider participants were ART providers at five healthcare facilities in four high HIV burden provinces in Thailand. Sample size calculation with 80% power and 95% participation rate yielded a target sample of 500 clients and 100 providers. Consecutive sampling was conducted according to the eligibility criteria to minimize sampling bias. All prospective participants were given information about the nature of the study, and those who agreed provided written informed consent. During the study, participants were informed that they could withdraw from the study at any time and their withdrawal would not affect the HIV services they received. All client participants received 300 baht (US$10) as travel compensation while provider participants did not receive any compensation.

The study was approved by the Chulalongkorn University Institutional Review Board (IRB: 109/60), Chiang Mai Provincial Public Health Office (cm0032.003.1/3327), and Research Ethics Committee of Hatyai Hospital (IRB: 35/2561).

### 2.2 Study sites

Thai Red Cross Anonymous Clinic is an HIV/sexually transmitted infection (STI) testing centre and a same‐day ART initiation hub located in the centre of Bangkok. It provides free HIV testing and same‐day ART initiation service, after which clients will be referred to their registered hospital according to their national health benefit scheme for long‐term ART maintenance. However, ART clients have the option to pay for treatment at the after‐hours clinic. TRCAC serves approximately 2,000 ART clients/year. Nakornping Hospital in Chiang Mai, Hatyai Hospital in Songkhla, and Queen Savang Vadhana Memorial Hospital in Chonburi are tertiary care hospitals that serve approximately 2800, 3000, and 4000 ART clients/year, respectively. Sarapee hospital in Chiang Mai is a secondary care hospital with approximately 500 ART clients/year. These hospitals have several ART maintenance options in addition to conventional care, including nurse‐led clinical consultation, pharmacist‐led or volunteer‐led, fast‐track ART refill, out‐of‐facility ART pickup centres led by pharmacists or community health workers, and ART home delivery via mail. These hospitals are under Thailand’s national health scheme system whereby secondary and tertiary care healthcare facilities provide ART maintenance services free‐of‐charge. These ART clinics are run by a team of nurses, navigators, community health workers, and/or health volunteers under the lead of the general or infectious disease physicians with the support from the facility’s pharmacists and medical technologists for ART dispensing and laboratory testing at the order of the physicians.

### 2.3 Data collection

Two sets of corresponding anonymous, self‐administered questionnaires in Thai were given to participating clients and providers to complete in approximately 15 minutes at the facilities. The questionnaires were divided into four sections: participant characteristics, service satisfaction (for clients only), attitudes towards DSD (for providers only), preferences for HIV services that they would like to receive, and expectations and concerns about DSD. There were 44 questions in the client version (Appendix S1) and 34 questions in the provider version (Appendix S2). The participants could choose to skip questions they wished not to answer.

### 2.4 Statistical analysis

After reviewing the entirety of the questionnaire results, questions pertinent to the study’s objective were included in the analysis. Descriptive statistics were used to summarize demographic characteristics, preferences, expectations and concerns of participants, and presented as percentages and 95% confidence intervals (CI) for categorical variables and as medians and interquartile ranges (IQR) for continuous variables. Sub‐population analyses were conducted to discern service preferences by age (<38 years vs. ≥38 years), assigned sex at birth, gender populations (general population, MSM, TGW and other), and study sites.

Statistical analysis was conducted with Stata version 15.0 (StataCorp, College Station, TX, USA).

## 3 RESULTS

A total of 500 clients and 52 providers completed the questionnaires. The majority of clients identified themselves as females (48.5%; 95% CI: 44.1 to 52.9), followed by males (31.3%; 95% CI: 27.4 to 35.6), MSM (16.8%; 95% CI: 13.7 to 20.3), and TGW (2.4%; 95% CI: 1.4 to 4.2). The median (IQR) age of clients was 38.6 (29.8 to 45.5) years and 77.0% (95% CI: 73.1 to 80.5) had secondary education or lower. Among providers, 78.9% (95% CI: 65.2 to 88.1) identified themselves as females, followed by males (11.5%; 95% CI: 5.1 to 23.9), TGW (7.7%; 95% CI: 2.8 to 19.3), and MSM (1.9%; 95% CI: 0.3 to 13.2). The median (IQR) age was 37.3 (27.3 to 45.1) years with 73.1% (95% CI: 59.0 to 83.1) having a bachelor’s degree and 7.7% (95% CI: 2.8 to 19.3) having a master’s degree or higher. All providers worked in the ART clinics as nurses (28.8%; 95% CI: 18.3 to 43.7), navigators (25.0%; 95% CI: 15.1 to 39.6), health volunteers (11.5%; 95% CI: 5.2 to 24.4), community health workers (9.6%; 95% CI: 4.0 to 22.0), physicians (3.8%; 95% CI: 0.9 to 15.0), pharmacists (3.8%; 95% CI: 0.9 to 15.0), medical technologists (3.8%; 95% CI: 0.9 to 15.0), and others (11.5%; 95% CI: 5.2 to 24.4) (Table 1).

**Table 1:** Demographic characteristics

	Overall		Clients		Providers	
	(N = 552)	95% CI	(N = 500)	95% CI	(N = 52)	95% CI
Assigned sex at birth, n (%)						
Male	273 (49.8)	(45.6, 54.0)	263 (52.9)	(48.5, 57.3)	10 (19.6)	(10.7, 33.3)
Female	275 (50.2)	(46.0, 54.4)	234 (47.1)	(42.7, 51.5)	41 (80.4)	(66.7, 89.3)
Identify oneself as, n (%)						
Male	161 (29.4)	(25.8, 33.4)	155 (31.3)	(27.4, 35.6)	6 (11.5)	(5.1, 23.9)
Female	281 (51.4)	(47.2, 55.6)	240 (48.5)	(44.1, 52.9)	41 (78.9)	(65.2, 88.1)
MSM	84 (15.4)	(12.6, 18.6)	83 (16.8)	(13.7, 20.3)	1 (1.9)	(0.3, 13.2)
TGW	16 (2.9)	(1.8, 4.7)	12 (2.4)	(1.4, 4.2)	4 (7.7)	(2.8, 19.3)
TGM	2 (0.4)	(0.1, 1.5)	2 (0.4)	(0.1, 1.6)	0 (0)	(0, 0)
Other	3 (0.6)	(0.2, 1.7)	3 (0.6)	(0.2, 1.9)	0 (0)	(0, 0)
Age (years)						
Median (IQR)	38.5 (29.7, 45.3)	–	38.6 (29.8, 45.5)	–	37.3 (27.3, 45.1)	–
Education level, n (%)						
Secondary school or lower	392 (71.6)	(67.6, 7.52)	382 (77.0)	(73.1, 80.5)	10 (19.2)	(10.4, 32.7)
Bachelor's degree	141 (25.7)	(22.2, 29.6)	103 (20.8)	(17.4, 24.6)	38 (73.1)	(59.0, 83.6)
Master's degree or higher	15 (2.7)	(1.7, 4.5)	11 (2.2)	(1.2, 4.0)	4 (7.7)	(2.8, 19.3)
Study sites, n (%)						
TRCAC	120 (21.7)	(18.5, 25.4)	100 (20.0)	(16.7, 23.8)	20 (38.5)	(26.0, 52.7)
QSV	110 (19.9)	(16.8, 23.5)	100 (20.0)	(16.7, 23.8)	10 (19.2)	(10.4, 32.7)
NKP	109 (19.7)	(16.6, 23.3)	100 (20.0)	(16.7, 23.8)	9 (17.3)	(9.1, 30.6)
SRP	111 (20.1)	(17.0, 23.7)	100 (20.0)	(16.7, 23.8)	11 (21.2)	(11.9, 34.8)
HY	102 (18.5)	(15.4, 21.9)	100 (20.0)	(16.7, 23.8)	2 (3.8)	(0.9, 14.7)
Position, n (%)						
Physician	–		–		2 (3.8)	(0.9, 15.0)
Nurse	–		–		15 (28.8)	(18.3, 43.7)
Pharmacist	–		–		2 (3.8)	(0.9, 15.0)
Medical technologist	–		–		2 (3.8)	(0.9, 15.0)
Community health worker	–		–		5 (9.6)	(4.0, 22.0)
Health volunteer	–		–		6 (11.5)	(5.2, 24.4)
Navigator	–		–		13 (25.0)	(15.1, 39.6)
Other	–		–		6 (11.5)	(5.2, 24.4)

95% CI, 95% confidence interval; HY, Hatyai Hospital; IQR, interquartile range; MSM, men who have sex with men; NKP, Nakornping Hospital; QSV, Queen Savang Vadhana Memorial Hospital; SRP, Sarapee Hospital; TGM, transgender men; TGW, transgender women; TRCAC, Thai Red Cross Anonymous Clinic.

### 3.1 Preferences

**Figure 1:**
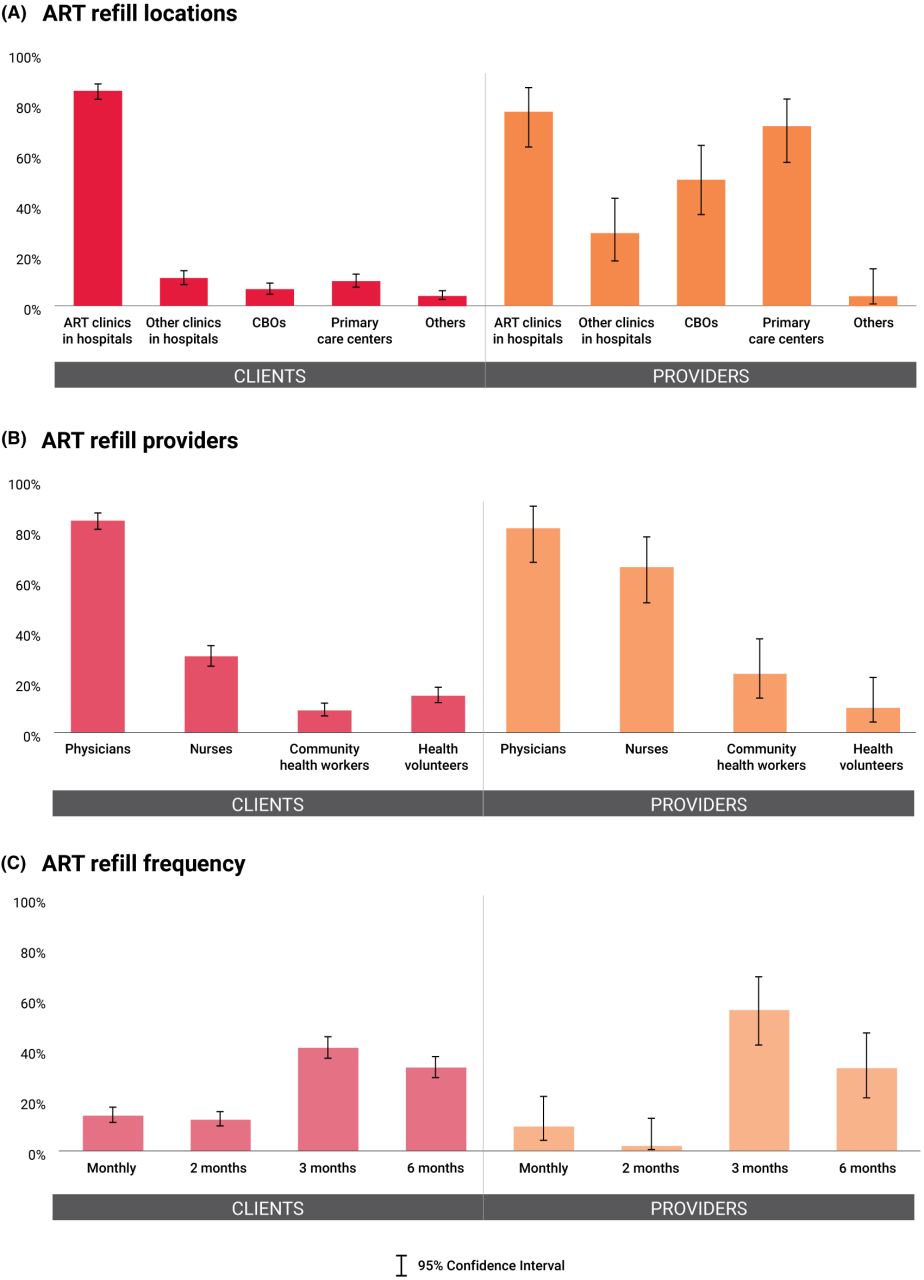
Preferences for antiretroviral therapy refill. Preferences for antiretroviral therapy (ART) refill in terms of **(A)** type of locations, **(B)** type of providers, and **(C)** frequency of provision among clients and providers. ART, antiretroviral therapy; CBOs, community‐based organizations.

**Figure 2:**
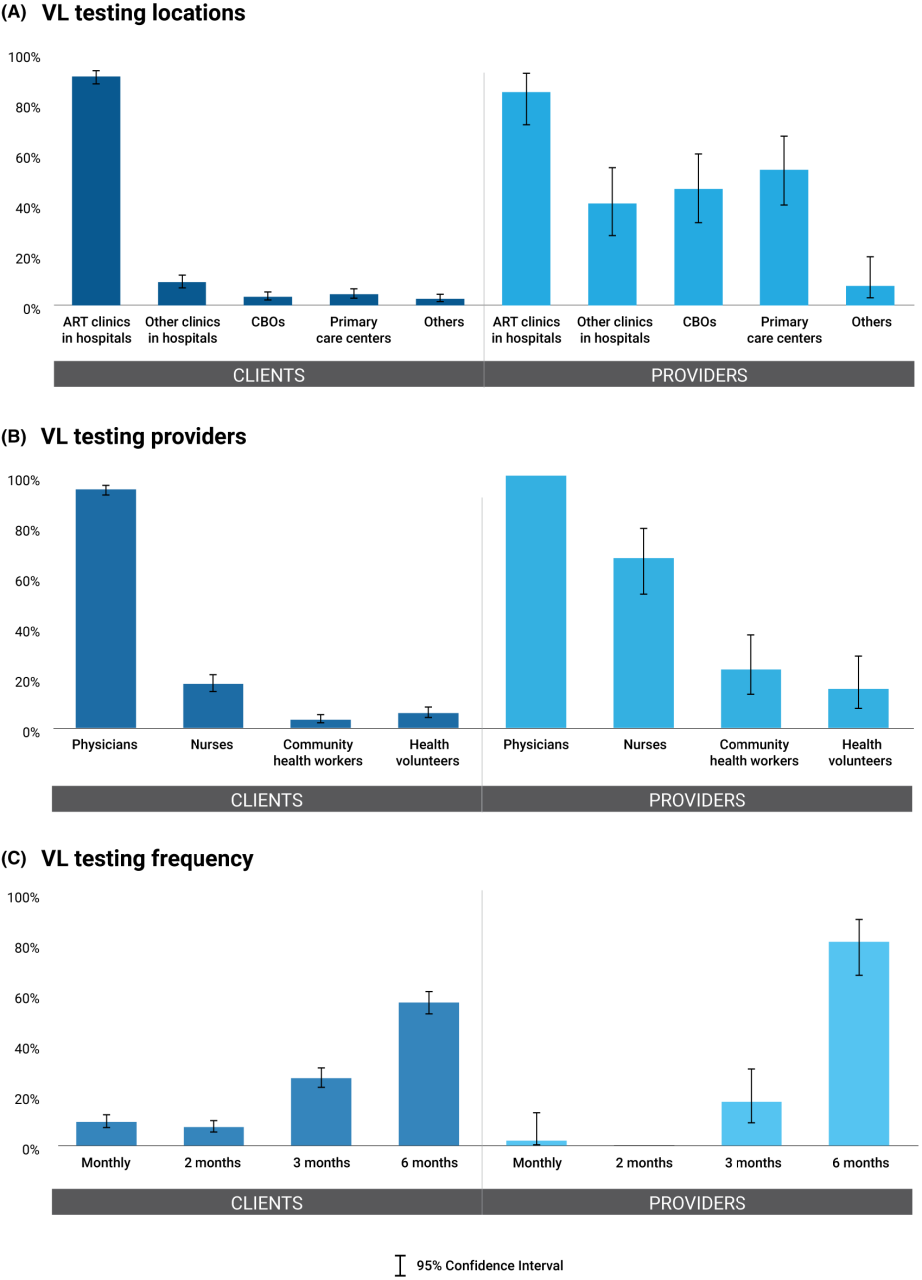
Preferences for viral load testing. Preferences for viral load (VL) testing in terms of **(A)** type of locations, **(B)** type of providers, and **(C)** frequency of provision among clients and providers. VL testing, viral load testing; CBOs, community‐based organizations.

Clients and providers were asked to rate their preferred locations, frequency, and providers for four components of ART maintenance services: ART refill, viral load (VL) testing, HIV/STI monitoring, and psychosocial support. ART clinics in hospitals were the most acceptable location for ART refill among clients (85.2%; 95% CI: 81.8 to 88.1) and providers (76.9%; 95% CI: 63.1 to 86.7), but many providers also indicated ART refill could be delivered through primary care centres (71.2%; 95% CI: 57.0 to 82.1) and CBOs (50%; 95% CI: 36.3 to 63.7) – a pattern that was identified across all four services. Most clients and providers would like physicians to dispense ART (83.8%; 95% CI: 80.3 to 86.8, vs. 80.8%; 95% CI: 67.3 to 89.6), followed by nurses (30.0%; 95% CI: 26.1 to 34.2, vs. 65.4%; 95% CI: 51.1 to 77.3). They also would like to refill ART every three months (40.8%; 95% CI: 36.6 to 45.2, vs. 55.8%; 95% CI: 41.7 to 69.0), followed by every six months (33.0%; 95% CI: 29.0 to 37.3, vs. 32.7%; 95% CI: 21.1 to 46.9) (Figure 1). Most clients and providers agreed that physicians should order VL testing (94.6%; 95% CI: 92.2 to 96.3, vs. 100.0%), and the majority preferred it to be done every six months (56.7%; 95% CI: 52.3 to 61.0, vs. 80.8%; 95% CI: 67.3 to 89.6) (Figure 2).

**Figure 3:**
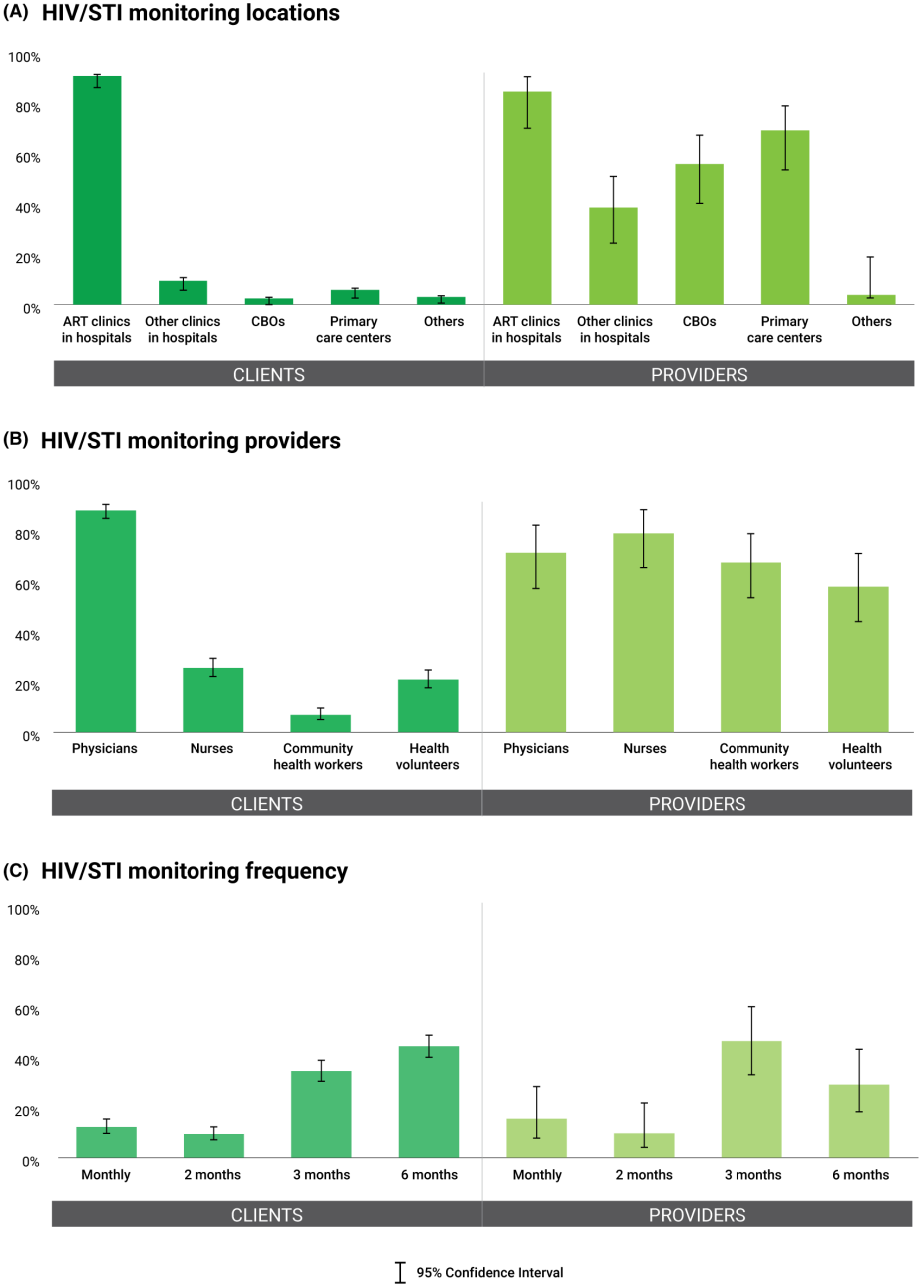
Preferences for HIV/sexually transmitted infection (STI) monitoring. Preferences for HIV/ sexually transmitted infection (STI) monitoring in terms of **(A)** type of locations, **(B)** type of providers, and **(C)** frequency of provision among clients and providers. STI, sexually transmitted infection; CBOs, community‐based organizations.

**Figure 4:**
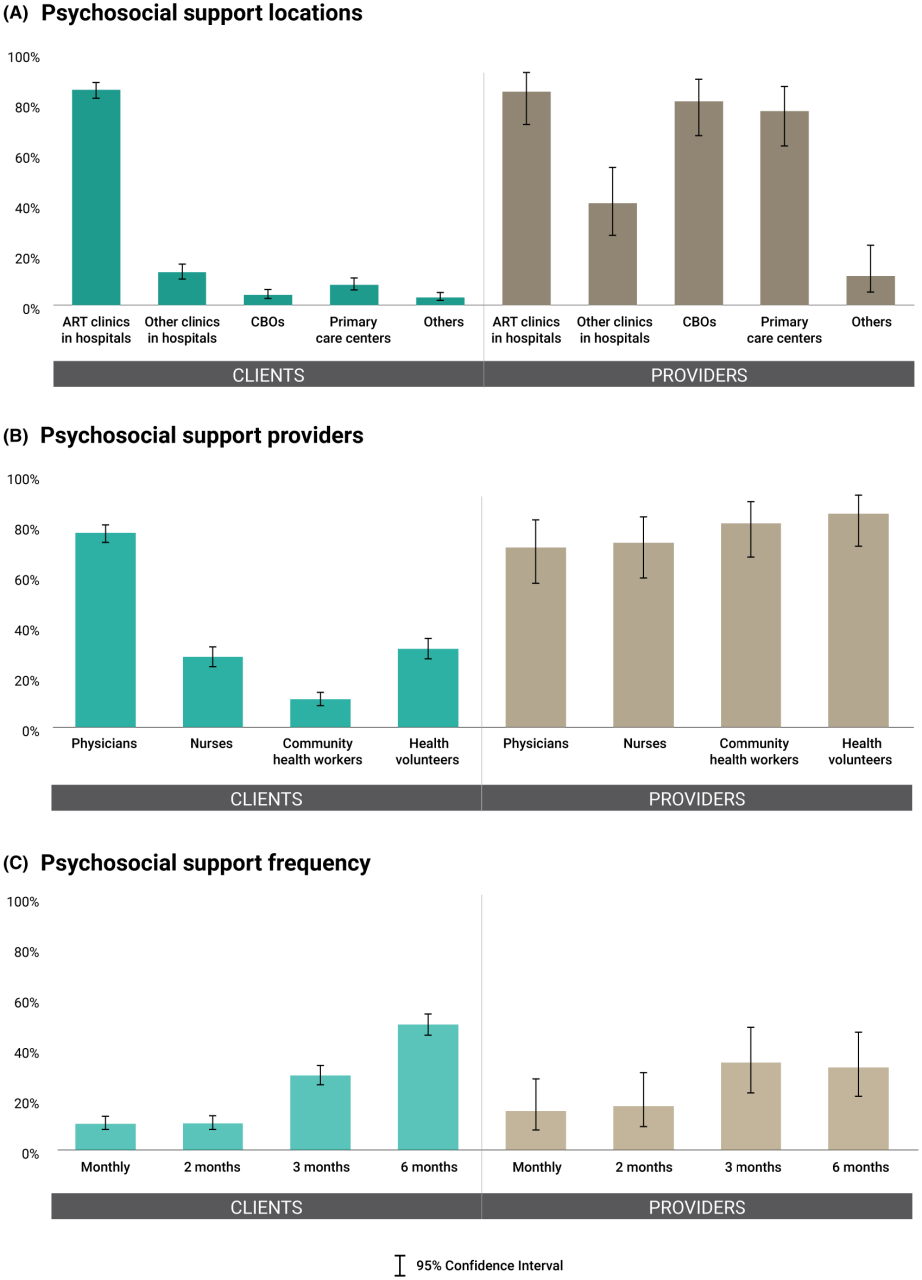
Preferences for psychosocial support. Preferences for psychosocial support in terms of **(A)** type of locations, **(B)** type of providers, and **(C)** frequency of provision among clients and providers. CBOs, community‐based organizations.

Figures 3 and 4 show that clients preferred physicians to provide HIV/STI monitoring (88.0%; 95% CI: 84.8 to 90.6) and psychosocial support (77.0%; 95% CI: 73.1 to 80.5). Interestingly, providers wanted nurses (78.9%; 95% CI: 65.2 to 88.1), followed by physicians (71.2%; 95% CI: 57.0 to 82.1), community health workers (67.3%; 95% CI: 53.1 to 78.9), and health volunteers (57.7%; 95% CI: 43.6 to 70.7) to monitor HIV/STIs, and wanted health volunteers (84.6%; 95% CI: 71.6 to 92.3), community health workers (80.8%; 95% CI: 67.3 to 89.6), nurses (73.1%; 95% CI: 59.0 to 83.6), and physicians (71.2%; 95% CI: 57.0 to 82.1) to provide psychosocial support. Nonetheless, the majority of both clients and providers agreed that these two services should be provided every three (29.5% to 46.2%) or six months (28.9% to 49.7%) (Table S1).

Sub‐population analyses found little differences between populations. More clients ≥38 years preferred to receive the following services from health volunteers than younger clients: ART refill (17.5%; 95% CI: 13.2 to 22.9, vs. 8.6%; 95% CI: 5.5 to 13.1), HIV/STI monitoring (24.6%; 95% CI: 19.5 to 30.5, vs. 10.9%; 95% CI: 7.4 to 15.7), and psychosocial support (37.5%; 95% CI: 31.6 to 43.8, vs. 19.0%; 95% CI: 14.3 to 24.7) (Table S3). Slight differences were found when the data were stratified by assigned sex at birth and gender populations. More assigned male providers preferred community health workers to provide psychosocial support than their female counterparts (100% vs. 24.4%; 95% CI: 13.3 to 40.3) (Table S4). Less general population clients preferred HIV/STI monitoring every two months more than MSM clients (7.2%; 95% CI: 5.0 to 10.2, vs. 18.1%; 95% CI: 11.2 to 27.9) (Table S5). Clients at TRCAC, secondary care hospitals and tertiary care hospitals had slightly different responses in preferences for other clinics in hospitals, community health workers, health volunteers, and service frequency (Table S6).

### 3.2 Expectation and concerns

**Figure 5:**
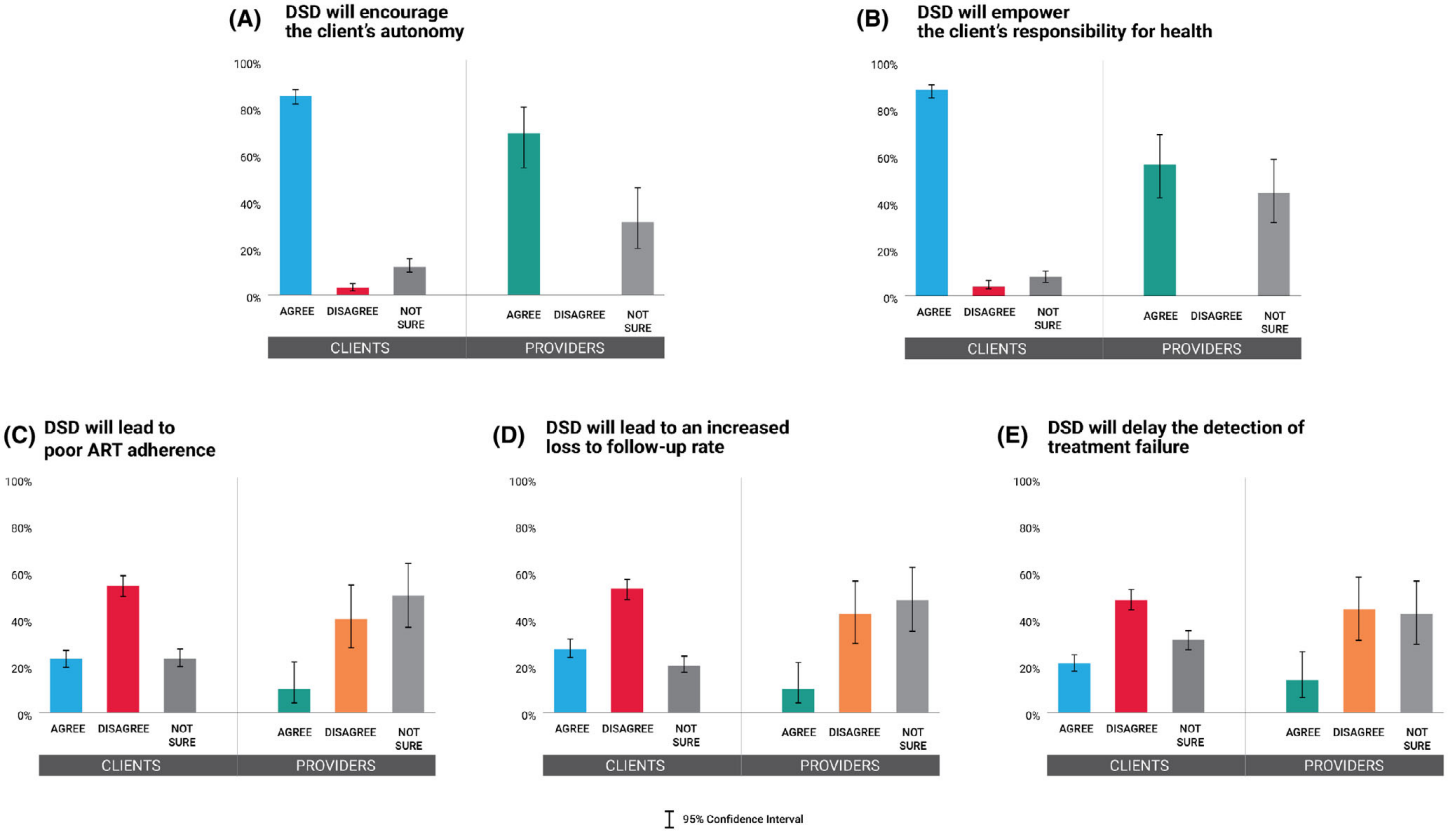
Expectations and concerns towards differentiated service delivery for antiretroviral therapy maintenance. Attitudes towards differentiated service delivery (DSD) among clients and providers whether DSD would **(A)** encourage the client’s autonomy, **(B)** empower the client’s responsibility for health, **(C)** lead to poor ART adherence, **(D)** lead to an increased loss to follow‐up rate, and **(E)** delay the detection of treatment failure. DSD, differentiated service delivery.

Figure 5 shows that the majority of clients and providers expected that differentiated ART maintenance service would encourage the client’s autonomy (84.9%; 95% CI: 81.4 to 87.8, vs. 68.6%; 95% CI: 54.3 to 80.1) and empower the client’s responsibility for their health (87.7%; 95% CI: 84.5 to 90.3, vs. 55.8%; 95% CI: 41.7 to 69.0). When asked about concerns, about half of both clients and providers disagreed that DSD would lead to poor ART retention (54.0%; 95% CI: 49.6 to 58.4, vs. 40.4%; 95% CI: 27.6 to 54.6), increased loss to follow‐up rates among clients (52.5%; 95% CI: 48.1 to 56.9, vs. 42.3%; 95% CI: 29.3 to 56.4), and delayed detection of treatment failure (48.3%; 95% CI: 43.9 to 52.7, vs. 44.2%; 95% CI: 31.0 to 58.3). However, 31.4% (95% CI: 19.9 to 45.7) and 44.2% (95% CI: 31.0 to 58.3) of providers were unsure about these expectations, and 50.0% (95% CI: 36.3 to 63.7), 48.1% (95% CI: 34.5 to 61.9), and 42.3 (95% CI: 29.3 to 56.4) of providers were unsure about these concerns, respectively (Table S2).

## 4 DISCUSSION

This is the first study to document the preferences regarding ART maintenance services among clients and providers of healthcare facilities in Thailand. More than three quarters of Thai clients preferred ART refill, VL testing, HIV/STI monitoring, and psychosocial support to be provided by physicians – 90% also wanted these services to happen in the hospitals. While more than 80% of clients felt that DSD would encourage their autonomy and empower them to take responsibility for health, up to 40% of providers were unsure about these expectations. Moreover, around half of providers were unsure if DSD would lead to increased loss to follow‐up, poor ART adherence and delayed detection of treatment failure.

Our finding shows that most clients have a strong preference for ART maintenance services at the ART clinic in hospitals, provided by physicians. Most clients and providers agreed to have less frequent visits for any ART maintenance services with more preferring to refill ART every three months than six months. In contrast, qualitative studies conducted in Malawi and Zambia found that most clients indicated six or twelve months, and most providers indicated six months as their ideal ART supply over three months as it coincided with clinical appointment [17, 18]. Their clients viewed that six‐month clinical appointment and ART dispensing reduced transport time and costs, increased time for income‐generating activities, and gave a sense of freedom and normalcy. For providers, it relieved workload, alleviated clinic congestion, and improved their ability to see unstable clients [17, 18]. However, a study conducted in Ethiopia found that some clients refused six‐month visit due to concern about ART storage, lack of assurance from frequent checkups and unsynchronized pickups with family members [19]. Hence, the preference for multi‐month refill is personal. Providers in a study conducted in South Africa also raised a concern about whether the existing drug supply system could accommodate six‐month ART refill at scale [20]. The strong preference for three‐month ART refill among our clients and providers might be explained by the fact that it is a common practice in Thailand. Despite the WHO recommending less frequent medication pickups up to every six months for stable clients [8], the lack of local recommendation [3] and supportive national ART stock reimbursement system [21] may hinder its adoption in Thailand. With the Guideline on Differentiated Care for Antiretroviral Treatment Service Delivery for Stable People Living with HIV in Thai Health Care Setting, which includes a recommendation for six‐month ART refill, released in March 2020 [22], six‐month ART refill is expected to become a more common practice in Thailand and gain clients and providers’ acceptance.

Decentralization opportunities are undermined by the low preference among our clients, despite some providers’ willingness. While more than half of our providers were supportive of services delivered through primary care centres, less than 10% of our clients preferred this location. The primary care centres have the potential to become ART dispensing sites as they are staffed with registered nurses and public health workers, and due to the established network to refer complex cases to secondary and tertiary care facilities under the Ministry of Public Health (MoPH) system. However, low preference for this location might be due to the fear of stigma and discrimination as the proximity of the primary care centre to the community might increase the risk of unintentional HIV status disclosure to neighbours, family members, and friends [5, 23]. Similarly, very few of our clients preferred CBOs, possibly because most of our clients identified as straight males and females. These clients might not see the need to attend CBOs as they did not face gender‐based stigma and discrimination that push the KP clients away from the conventional services [24, 25, 26]. Nonetheless, since the majority of our participants viewed that the ability to meet client demands will likely impact the client response to HIV care, ART programme implementers and policymakers should explore options to adapt the services to optimize client preferences while not overlooking the preferences of a few or marginalized clients. This may be done by offering client‐targeted, desired services. Other novel decentralization strategies, such as telehealth with mail/bike delivery of ART incorporated into physician‐led, facility‐based ART maintenance model, should be explored.

Task shifting to non‐physician healthcare providers was met with client and provider resistance. Clients may feel discouraged to participate in the decision‐making process regarding their own health due to the socio‐cultural values that put an emphasis on respect for authority, social hierarchy, and compliance with physician orders without questioning [27, 28, 29]. This may cause clients to develop a dependent relationship with physicians and explain our clients’ continuous preference to see physicians during routine follow‐up visits. Most providers also only accepted community health workers and health volunteers to monitor HIV/STI and provide psychosocial support, but not to distribute ART or order routine VL testing. This implies concerns about the capacity of lower cadre providers and the potential for task‐shifting to result in lower quality clinical care. In Zambia, a comprehensive and structured approach for task‐shifted ART maintenance tailored to each cadre of health providers was rolled out and proven to allow the expansion of treatment access without compromising the quality of care [30]. In Thailand, a similar training and certification platform is being offered to empower and equip KP lay providers with the necessary knowledge and skills to provide services across the HIV prevention and treatment cascade [31, 32]. This platform must be scaled up to optimize the contribution of lay providers in supporting HIV services at the country level. Successful task shifting will necessitate the engagement of governmental agencies to recognize the training platform and the expanded duties of non‐physician providers via certification, legal support, and incorporation into policy guidance as well as a societal and cultural norm in order to make task shifting acceptable to clients and providers alike.

This study has several limitations. This study was conducted at five healthcare facilities in four provinces in Thailand, which might limit generalizability to clients and providers from other demographics and settings, such as primary care centres and CBOs. The data on clinical conditions and ART maintenance service models the clients received were not collected. The sub‐population analyses were conducted to further understand the participant preferences. The provider target sample size was not met as all available staff already completed the questionnaire during the study period. Though the majority of providers were nurses and navigators, and only two physicians and two pharmacists participated, this reflected the staff composition at Thai facilities. The question regarding VL testing frequency had choices up to only six months despite the national health benefit packages allow VL testing every six to twelve months for new and unstable clients and every 12 months for clients with undetectable VL. Due to the lack of literature, we had limited knowledge regarding the availability of ART maintenance models in Thailand and we framed our questionnaires according to the global DSD framework with no formative work done to validate the questionnaires. Nonetheless, this was the first study that asked and provided the basis for understanding the preferences of Thai clients and providers relating to ART maintenance. Further research is needed to better characterize needs and preferences among different cohorts of clients using validated toolsets. The cross‐sectional nature of the study may not reflect the changing needs and preferences of clients as their life and disease progress as well as the changed reality of health service delivery in the post‐COVID‐19 period. Future studies should examine the availability of the physician‐led, facility‐based ART maintenance model that was preferred by participants in this study, and assess its long‐term effectiveness to drive evidence‐based policy change to expand its practice as it is what Thai clients and providers demand.

## 5 CONCLUSIONS

Most of our clients and providers expressed a strong preference for ART maintenance services at the ART clinic in hospitals, provided by physicians, with less frequent visits up to every three or six months. This identified the physician‐led, facility‐based ART maintenance approach, such as clinical consultation and ART refill visit spacing, as one promising DSD model for the Thai setting. However, strong governmental endorsement through supportive guidelines and a national health scheme system is needed for the widespread implementation of the multi‐month ART refill, especially the six‐month ART refill. Furthermore, low preference for decentralization and task shifting, especially among clients, may prove challenging for implementing other DSD models that will alleviate the growing burden on PLHIV and the healthcare system. Collaborative efforts of governmental agencies, policymakers, health administrators, service providers, and PLHIV are required to advocate and legitimize decentralization, task shifting, and visit spacing.

## COMPETING INTERESTS

All authors declare no competing interests related to this work.

## AUTHORS’ CONTRIBUTIONS

SL drafted the original manuscript. SL, SA and RR developed the analysis plan. TS, PS and NP designed and directed the study. TC, ST, PM, PL, AN and AN led data collection at their respective sites. SL, JP and PM analysed the data. All authors critically reviewed the manuscript. SL and SA revised the manuscript according to comments received. PP, RR and NP approved the final version of the manuscript.

## Supporting information


**Table S1.** Service preferences

**Table S2**. Expectations and concerns towards DSD for ART maintenance

**Table S3**. Service preferences by age.

**Table S4**. Service preferences by assigned sex at birth

**Table S5**. Service preferences by populations

**Table S6**. Service preferences by study sites

**Appendix S1**. Client and provider preferences for HIV care questionnaire: client version.
**Appendix S2**. Client and provider preferences for HIV care questionnaire: provider version.
