# Supplementary material for: Client and provider preferences for HIV care: Implications for implementing differentiated service delivery in Thailand
Source: J Int AIDS Soc. 2021 Mar 31;24(4):e25693. doi: 10.1002/jia2.25693 (PMC8013790; doi:10.1002/jia2.25693)
Supplement: Supplementary file 1 — Table S1. Service preferences [file JIA2-24-e25693-s003.docx]

**S1 Table. Service preferences.**

|  | **Overall** |  | **Clients** |  | **Providers** |  |
| --- | --- | --- | --- | --- | --- | --- |
|  | **(N=552)**  **n (%)** | **95% CI** | **(N=500)**  **n (%)** | **95% CI** | **(N=52)**  **n (%)** | **95% CI** |
| **ART refill locations (more than one answer possible)** |  |  |  |  |  |  |
| ART clinics in hospitals | 466 (84.4) | (81.1, 87.2) | 426 (85.2) | (81.8, 88.1) | 40 (76.9) | (63.1, 86.7) |
| Other clinics in hospitals | 70 (12.7) | (10.1, 15.7) | 55 (11.0) | (8.5, 14.1) | 15 (28.9) | (17.9, 43.0) |
| CBOs | 59 (10.7) | (8.4, 13.6) | 33 (6.6) | (4.7, 9.1) | 26 (50.0) | (36.3, 63.7) |
| Primary care centers | 86 (15.6) | (12.8, 18.9) | 49 (9.8) | (7.5, 12.7) | 37 (71.2) | (57.0, 82.1) |
| Others | 22 (4.0) | (2.6, 6.0) | 20 (4.0) | (2.6, 6.1) | 2 (3.9) | (0.9, 14.7) |
| **ART refill providers (more than one answer possible)** |  |  |  |  |  |  |
| Physicians | 461 (83.5) | (80.2, 86.4) | 419 (83.8) | (80.3, 86.8) | 42 (80.8) | (67.3, 89.6) |
| Nurses | 184 (33.3) | (29.5, 37.4) | 150 (30.0) | (26.1, 34.2) | 34 (65.4) | (51.1, 77.3) |
| Community health workers | 55 (10.0) | (7.7, 12.8) | 43 (8.6) | (6.4, 11.4) | 12 (23.1) | (13.3, 36.9) |
| Health volunteers | 77 (14.0) | (11.3, 17.1) | 72 (14.4) | (11.6, 17.8) | 5 (9.6) | (3.9, 21.6) |
| **ART refill frequency** |  |  |  |  |  |  |
| Monthly | 74 (13.5) | (10.9, 16.6) | 69 (13.9) | (11.1, 17.2) | 5 (9.6) | (3.9, 21.6) |
| 2 months | 62 (11.3) | (8.9, 14.2) | 61 (12.3) | (9.7, 15.5) | 1 (1.9) | (0.3, 13.2) |
| 3 months | 232 (42.2) | (38.2, 46.4) | 203 (40.8) | (36.6, 45.2) | 29 (55.8) | (41.7, 69.0) |
| 6 months | 181 (33.0) | (29.2, 37.0) | 164 (33.0) | (29.0, 37.3) | 17 (32.7) | (21.1, 46.9) |
| **VL testing locations (more than one answer possible)** |  |  |  |  |  |  |
| ART clinics in hospitals | 498 (90.2) | (87.4, 92.4) | 454 (90.8) | (87.9, 93.0) | 44 (84.6) | (71.6, 92.3) |
| Other clinics in hospitals | 67 (12.1) | (9.7, 15.1) | 46 (9.2) | (7.0, 12.1) | 21 (40.4) | (27.6, 54.6) |
| CBOs | 41 (7.4) | (5.5, 9.9) | 17 (3.4) | (2.1, 5.4) | 24 (46.2) | (32.8, 60.1) |
| Primary care centers | 50 (9.1) | (6.9, 11.8) | 22 (4.4) | (2.9, 6.6) | 28 (53.9) | (39.9, 67.2) |
| Others | 17 (3.1) | (1.9, 4.9) | 13 (2.6) | (0.015, 0.044) | 4 (7.7) | (2.8, 19.3) |
| **VL testing providers (more than one answer possible)** |  |  |  |  |  |  |
| Physicians | 525 (95.1) | (93.0, 96.6) | 473 (94.6) | (92.2, 96.3) | 52 (100.0) | - |
| Nurses | 122 (22.1) | (18.8, 25.8) | 87 (17.4) | (14.3, 21.0) | 35 (67.3) | (53.1, 78.9) |
| Community health workers | 28 (5.1) | (3.5, 7.3) | 16 (3.2) | (2.0, 5.2) | 12 (23.1) | (13.3, 36.9) |
| Health volunteers | 37 (6.7) | (4.9, 9.1) | 29 (5.8) | (4.1, 8.2) | 8 (15.4) | (7.7, 28.4) |
| **VL testing frequency** |  |  |  |  |  |  |
| Monthly | 47 (8.6) | (6.5, 11.3) | 46 (9.3) | (7.0, 12.2) | 1 (1.9) | (0.3, 13.2) |
| 2 months | 36 (6.6) | (4.8, 9.0) | 36 (7.3) | (5.3, 9.9) | 0 (0) | (0, 0) |
| 3 months | 141 (25.8) | (22.3, 29.7) | 132 (26.7) | (23.0, 30.8) | 9 (17.3) | (9.1, 30.6) |
| 6 months | 322 (59.0) | (54.8, 63.0) | 280 (56.7) | (52.3, 61.0) | 42 (80.8) | (67.3, 89.6) |
| **HIV/STI monitoring locations (more than one answer possible)** |  |  |  |  |  |  |
| ART clinics in hospitals | 498 (90.2) | (87.4, 92.4) | 454 (90.8) | (87.9, 93.0) | 44 (84.6) | (71.6, 92.3) |
| Other clinics in hospitals | 67 (12.1) | (9.7, 15.1) | 47 (9.4) | (7.1, 12.3) | 20 (38.5) | (26.0, 52.7) |
| CBOs | 41 (7.4) | (5.5, 9.9) | 12 (2.4) | (1.4, 4.2) | 29 (55.8) | (41.7, 69.0) |
| Primary care centers | 65 (11.8) | (9.3, 14.8) | 29 (5.8) | (4.1, 8.2) | 36 (69.2) | (55.0, 80.5) |
| Others | 17 (3.1) | (1.9, 4.9) | 15 (3.0) | (1.8, 4.9) | 2 (3.9) | (0.9, 14.7) |
| **HIV/STI monitoring providers (more than one answer possible)** |  |  |  |  |  |  |
| Physicians | 477 (86.4) | (83.3, 89.0) | 440 (88.0) | (84.8, 90.6) | 37 (71.2) | (57.0, 82.1) |
| Nurses | 168 (30.4) | (26.7, 34.4) | 127 (25.4) | (21.8, 29.4) | 41 (78.9) | (65.2, 88.1) |
| Community health workers | 69 (12.5) | (10.0, 15.5) | 34 (6.8) | (4.9, 9.4) | 35 (67.3) | (53.1, 78.9) |
| Health volunteers | 134 (24.3) | (20.9, 28.0) | 104 (20.8) | (17.5, 24.6) | 30 (57.7) | (43.6, 70.7) |
| **HIV/STI monitoring frequency** |  |  |  |  |  |  |
| Monthly | 68 (12.4) | (9.9, 15.5) | 60 (12.1) | (9.5, 15.3) | 8 (15.4) | (7.7, 28.4) |
| 2 months | 51 (9.3) | (7.2, 12.1) | 46 (9.3) | (7.0, 12.2) | 5 (9.6) | (3.9, 21.6) |
| 3 months | 194 (35.5) | (31.6, 39.6) | 170 (34.3) | (30.3, 38.7) | 24 (46.2) | (32.8, 60.1) |
| 6 months | 234 (42.8) | (38.7, 47.0) | 219 (44.2) | (39.9, 48.7) | 15 (28.9) | (17.9, 43.0) |
| **Psychosocial support locations (more than one answer possible)** |  |  |  |  |  |  |
| ART clinics in hospitals | 471 (85.3) | (82.1, 88.0) | 427 (85.4) | (82.0, 88.2) | 44 (84.6) | (71.6, 92.3) |
| Other clinics in hospitals | 86 (15.6) | (12.8, 18.9) | 65 (13.0) | (10.3, 16.3) | 21 (40.4) | (27.6, 54.6) |
| CBOs | 62 (11.2) | (8.8, 14.2) | 20 (4.0) | (2.6, 6.1) | 42 (80.8) | (67.3, 89.6) |
| Primary care centers | 80 (14.5) | (11.8, 17.7) | 40 (8.0) | (5.9, 10.7) | 40 (76.9) | (63.1, 86.7) |
| Others | 21 (3.8) | (2.5, 5.8) | 15 (3.0) | (1.8, 4.9) | 6 (11.5) | (5.1, 23.9) |
| **Psychosocial support providers (more than one answer possible)** |  |  |  |  |  |  |
| Physicians | 422 (76.5) | (72.7, 79.8) | 385 (77.0) | (73.1, 80.5) | 37 (71.2) | (57.0, 82.1) |
| Nurses | 177 (32.1) | (28.3, 36.1) | 139 (27.8) | (24.0, 31.9) | 38 (73.1) | (59.0, 83.6) |
| Community health workers | 97 (17.6) | (14.6, 21.0) | 55 (11.0) | (8.5, 14.1) | 42 (80.8) | (67.3, 89.6) |
| Health volunteers | 199 (36.1) | (32.1, 40.2) | 155 (31.0) | (27.1, 35.2) | 44 (84.6) | (71.6, 92.3) |
| **Psychosocial support frequency** |  |  |  |  |  |  |
| Monthly | 59 (10.8) | (8.4, 13.7) | 51 (10.3) | (7.9, 13.3) | 8 (15.4) | (7.7, 28.4) |
| 2 months | 61 (11.2) | (8.8, 14.1) | 52 (10.5) | (8.1, 13.5) | 9 (17.3) | (9.1, 30.6) |
| 3 months | 164 (30.0) | (26.3, 34.0) | 146 (29.5) | (25.6, 33.7) | 18 (34.6) | (22.7, 48.9) |
| 6 months | 263 (48.1) | (43.9, 52.3) | 246 (49.7) | (45.3, 54.1) | 17 (32.7) | (21.1, 46.9) |

95% CI, 95% confidence interval; ART, antiretroviral therapy; CBOs, community-based organizations; VL, viral load; STI, sexually transmitted infection.
