# Supplementary material for: Client and provider preferences for HIV care: Implications for implementing differentiated service delivery in Thailand
Source: J Int AIDS Soc. 2021 Mar 31;24(4):e25693. doi: 10.1002/jia2.25693 (PMC8013790; doi:10.1002/jia2.25693)
Supplement: Supplementary file 2 — Table S2. Expectations and concerns towards DSD for ART maintenance [file JIA2-24-e25693-s001.docx]

**S2 Table. Expectations and concerns towards differentiated service delivery for antiretroviral therapy maintenance.**

|  | **Overall** | |  | **Clients** | |  | **Providers** | |  |
| --- | --- | --- | --- | --- | --- | --- | --- | --- | --- |
|  | **(N=552) n (%)** | **95% CI** | | **(N=500) n (%)** | **95% CI** | | **(n=52)**  **n (%)** | **95% CI** | |
| **Expectations** |  |  | |  |  | |  |  | |
| **DSD** **would encourage the client’s autonomy** |  |  | |  |  | |  |  | |
| Agree | 456 (83.4) | (80.0, 86.3) | | 421 (84.9) | (81.4, 87.8) | | 35 (68.6) | (54.3, 80.1) | |
| Disagree | 14 (2.7) | (1.5, 4.3) | | 14 (2.8) | (1.7, 4.7) | | 0 (0) | (0, 0) | |
| Not sure | 77 (14.1) | (11.4, 17.3) | | 61 (12.3) | (9.7, 15.5) | | 16 (31.4) | (19.9, 45.7) | |
| **DSD would empower the client’s responsibility for health** |  |  | |  |  | |  |  | |
| Agree | 464 (84.7) | (81.4, 87.5) | | 435 (87.7) | (84.5, 90.3) | | 29 (55.8) | (41.7, 69.0) | |
| Disagree | 22 (4.0) | (2.7, 6.0) | | 22 (4.4) | (2.9, 6.7) | | 0 (0) | (0, 0) | |
| Not sure | 62 (11.3) | (8.9, 14.3) | | 39 (7.9) | (5.8, 10.6) | | 23 (44.2) | (31.0, 58.3) | |
| **Concerns** |  |  | |  |  | |  |  | |
| **DSD would lead to poor ART adherence** |  |  | |  |  | |  |  | |
| Agree | 117 (21.5) | (18.2, 25.2) | | 112 (22.8) | (19.3, 26.7) | | 5 (9.6) | (3.9, 21.6) | |
| Disagree | 287 (52.8) | (48.5, 56.9) | | 266 (54.0) | (49.6, 58.4) | | 21 (40.4) | (27.6, 54.6) | |
| Not sure | 140 (25.7) | (22.2, 29.6) | | 114 (23.2) | (19.6, 27.1) | | 26 (50.0) | (36.3, 63.7) | |
| **DSD would lead to an increased loss to follow-up rate** |  |  | |  |  | |  |  | |
| Agree | 140 (25.5) | (22.0, 29.3) | | 135 (27.2) | (23.4, 31.3) | | 5 (9.6) | (3.9, 21.6) | |
| Disagree | 283 (51.5) | (47.4, 55.7) | | 261 (52.5) | (48.1, 56.9) | | 22 (42.3) | (29.3, 56.4) | |
| Not sure | 126 (23.0) | (19.6, 26.7) | | 101 (20.3) | (17.0, 24.1) | | 25 (48.1) | (34.5, 61.9) | |
| **DSD would delay the detection of treatment failure** |  |  | |  |  | |  |  | |
| Agree | 110 (20.2) | (17.0, 23.8) | | 103 (20.9) | (17.5, 24.7) | | 7 (13.5) | (6.4, 26.2) | |
| Disagree | 261 (47.9) | (43.7, 52.1) | | 238 (48.3) | (43.9, 52.7) | | 23 (44.2) | (31.0, 58.3) | |
| Not sure | 174 (31.9) | (28.1, 36.0) | | 152 (30.8) | (26.9, 35.1) | | 22 (42.3) | (29.3, 56.4) | |

95% CI, 95% confidence interval; DSD, differentiated service delivery; ART, antiretroviral therapy.
