# Supplementary material for: Client and provider preferences for HIV care: Implications for implementing differentiated service delivery in Thailand
Source: J Int AIDS Soc. 2021 Mar 31;24(4):e25693. doi: 10.1002/jia2.25693 (PMC8013790; doi:10.1002/jia2.25693)
Supplement: Supplementary file 3 — Table S3. Service preferences by age. [file JIA2-24-e25693-s005.docx]

**S3 Table. Service preferences by age.**

|  | **Clients** | | | | | **Providers** | | | |
| --- | --- | --- | --- | --- | --- | --- | --- | --- | --- |
|  | **Age <38 (N=221)**  **n (%)** | **95% CI** | **Age ≥38 (N=240)**  **n (%)** | **95% CI** | **Age <38 (N=22)**  **n (%)** | | **95% CI** | **Age ≥38 (N=22)**  **n (%)** | **95% CI** |
| **ART refill locations (more than one answer possible)** |  |  |  |  |  | |  |  |  |
| ART clinics in hospitals | 197 (89.1) | (84.3, 92.6) | 198 (82.5) | (77.2, 86.8) | 15 (68.2) | | (45.4, 84.6) | 18 (81.8) | (59.0, 93.4) |
| Other clinics in hospitals | 14 (6.3) | (3.8, 10.4) | 29 (12.1) | (8.5, 16.9) | 6 (27.3) | | (12.3, 50.2) | 6 (27.3) | (12.3, 50.2) |
| CBOs | 9 (4.1) | (2.1, 7.7) | 20 (8.3) | (5.4, 12.6) | 14 (63.6) | | (41.2, 81.4) | 10 (45.5) | (25.6, 66.9) |
| Primary care centers | 16 (7.2) | (4.5, 11.5) | 26 (10.8) | (7.5, 15.5) | 18 (81.8) | | (59.0, 93.4) | 14 (63.6) | (41.2, 81.4) |
| Others | 4 (1.8) | (6.8, 4.7) | 11 (4.6) | (2.5, 8.1) | 0 | | - | 2 (9.1) | (2.1, 31.6) |
| **ART refill providers (more than one answer possible)** |  |  |  |  |  | |  |  |  |
| Physicians | 180 (81.4) | (75.7, 86.1) | 203 (84.6) | (79.4, 88.6) | 17 (77.3) | | (54.3, 90.7) | 19 (86.4) | (63.7, 95.8) |
| Nurses | 77 (34.8) | (28.8, 41.4) | 60 (25.0) | (19.9, 30.9) | 12 (54.5) | | (33.1, 74.4) | 17 (77.3) | (54.3, 90.7) |
| Community health workers | 11 (5.0) | (2.8, 8.8) | 23 (9.6) | (6.4, 14.0) | 6 (27.3) | | (12.3, 50.2) | 5 (22.7) | (9.3, 45.7) |
| Health volunteers | 19 (8.6) | (5.5, 13.1) | 42 (17.5) | (13.2, 22.9) | 0 | | - | 4 (18.2) | (6.6, 41.0) |
| **ART refill frequency** |  |  |  |  |  | |  |  |  |
| Monthly | 27 (12.3) | (8.6, 17.4) | 35 (14.6) | (10.7, 19.7) | 1 (4.5) | | (0.6, 28.3) | 3 (13.6) | (4.2, 36.3) |
| 2 months | 35 (16.0) | (11.7, 21.5) | 19 (7.9) | (5.1, 12.2) | 0 | | - | 0 | - |
| 3 months | 74 (33.8) | (27.8, 40.3) | 112 (46.9) | (40.6, 53.2) | 13 (59.1) | | (37.1, 78.0) | 12 (54.5) | (33.1, 74.4) |
| 6 months | 83 (37.9) | (31.7, 44.5) | 73 (30.5) | (25.0, 36.7) | 8 (36.4) | | (18.6, 58.8) | 7 (31.8) | (15.4, 54.6) |
| **VL testing locations (more than one answer possible)** |  |  |  |  |  | |  |  |  |
| ART clinics in hospitals | 213 (96.4) | (92.9, 98.2) | 211 (87.9) | (83.1, 91.5) | 18 (81.8) | | (59.0, 93.4) | 19 (86.4) | (63.7, 95.8) |
| Other clinics in hospitals | 8 (3.6) | (1.8, 7.1) | 24 (10.0) | (6.8, 14.5) | 9 (40.9) | | (22.0, 62.9) | 9 (40.9) | (22.0, 62.9) |
| CBOs | 5 (2.3) | (0.9, 5.3) | 10 (4.2) | (2.2, 7.6) | 9 (40.9) | | (22.0, 62.9) | 12 (54.5) | (33.1, 74.4) |
| Primary care centers | 6 (2.7) | (1.2, 5.9) | 10 (4.2) | (2.2, 7.6) | 14 (63.4) | | (41.2, 81.4) | 12 (54.5) | (33.1, 74.4) |
| Others | 1 (0.5) | (0.1, 3.2) | 8 (3.3) | (1.7, 6.5) | 1 (4.5) | | (0.6, 28.3) | 2 (9.1) | (2.1, 31.6) |
| **VL testing providers (more than one answer possible)** |  |  |  |  |  | |  |  |  |
| Physicians | 206 (93.2) | (89.0, 95.9) | 228 (95.0) | (91.4, 97.1) | 22 (100) | | - | 22 (100) | - |
| Nurses | 41 (18.6) | (13.9, 24.3) | 37 (15.4) | (11.4, 20.6) | 10 (45.5) | | (25.6, 66.9) | 18 (81.8) | (59.0, 93.4) |
| Community health workers | 2 (0.9) | (0.2, 3.6) | 10 (4.2) | (2.2, 7.6) | 5 (22.7) | | (9.3, 45.7) | 5 (22.7) | (9.3, 45.7) |
| Health volunteers | 5 (2.3) | (0.9, 5.3) | 19 (7.9) | (5.1, 12.1) | 3 (13.6) | | (4.2, 36.3) | 3 (13.6) | (4.2, 36.3) |
| **VL testing frequency** |  |  |  |  |  | |  |  |  |
| Monthly | 11 (5.1) | (2.8, 9.0) | 23 (9.6) | (6.5, 14.1) | 0 | | - | 1 (4.5) | (0.6, 28.3) |
| 2 months | 19 (8.8) | (5.7, 13.4) | 11 (4.6) | (2.6, 8.1) | 0 | | - | 0 | - |
| 3 months | 60 (27.8) | (22.2, 34.2) | 59 (24.7) | (19.6, 30.6) | 3 (13.6) | | (4.2, 36.3) | 4 (18.2) | (6.6, 41.0) |
| 6 months | 126 (58.3) | (51.6, 64.8) | 146 (61.1) | (54.7, 67.1) | 19 (86.4) | | (63.7, 95.8) | 17 (77.3) | (54.3, 90.7) |
| **HIV/STI monitoring locations (more than one answer possible)** |  |  |  |  |  | |  |  |  |
| ART clinics in hospitals | 213 (96.4) | (92.9, 98.2) | 210 (87.5) | (82.7, 91.1) | 17 (77.3) | | (54.3, 90.7) | 19 (86.4) | (63.7, 95.8) |
| Other clinics in hospitals | 13 (5.9) | (3.4, 9.9) | 25 (10.4) | (7.1, 15.0) | 10 (45.5) | | (25.6, 66.9) | 7 (31.8) | (15.4, 54.6) |
| CBOs | 3 (1.4) | (0.4, 4.1) | 7 (2.9) | (1.4, 6.0) | 13 (59.1) | | (37.1, 78.0) | 13 (59.1) | (37.1, 78.0) |
| Primary care centers | 7 (3.2) | (1.5, 6.5) | 18 (7.5) | (4.8, 11.6) | 17 (77.3) | | (54.3, 90.7) | 15 (68.2) | (45.4, 84.6) |
| Others | 1 (0.5) | (0.1, 3.2) | 10 (4.2) | (2.2, 7.6) | 0 | | - | 2 (9.1) | (2.1, 31.6) |
| **HIV/STI monitoring providers (more than one answer possible)** |  |  |  |  |  | |  |  |  |
| Physicians | 197 (89.1) | (84.3, 92.6) | 211 (87.9) | (83.1, 91.5) | 17 (77.3) | | (54.3, 90.7) | 16 (72.7) | (49.8, 87.8) |
| Nurses | 62 (28.1) | (22.5, 34.4) | 51 (21.3) | (16.5, 26.9) | 16 (77.3) | | (54.3, 90.7) | 17 (72.7) | (49.8, 87.8) |
| Community health workers | 11 (5.0) | (2.8, 8.8) | 16 (6.7) | (4.1, 10.6) | 13 (59.1) | | (37.1, 78.0) | 15 (68.2) | (45.4, 84.6) |
| Health volunteers | 24 (10.9) | (7.4, 15.7) | 59 (24.6) | (19.5, 30.5) | 9 (40.9) | | (22.0, 62.9) | 15 (68.2) | (45.4, 84.6) |
| **HIV/STI monitoring frequency** |  |  |  |  |  | |  |  |  |
| Monthly | 19 (8.7) | (5.6, 13.3) | 27 (11.3) | (7.9, 16.1) | 1 (4.5) | | (0.6, 28.3) | 7 (31.8) | (15.4, 54.6) |
| 2 months | 27 (12.4) | (8.6, 17.5) | 16 (6.7) | (4.1, 10.7) | 3 (13.6) | | (4.2, 36.3) | 0 | - |
| 3 months | 66 (30.3) | (24.5, 36.7) | 89 (37.4) | (31.5, 43.7) | 10 (45.5) | | (25.6, 66.9) | 9 (40.9) | (22.0, 62.9) |
| 6 months | 106 (48.6) | (42.0, 55.3) | 106 (44.5) | (38.3, 50.9) | 8 (36.4) | | (18.6, 58.8) | 6 (27.3) | (12.3, 50.2) |
| **Psychosocial support locations (more than one answer possible)** |  |  |  |  |  | |  |  |  |
| ART clinics in hospitals | 205 (92.8) | (88.5, 98.6) | 193 (80.4) | (74.9, 85.0) | 18 (81.8) | | (59.0, 93.4) | 19 (86.4) | (63.7, 95.8) |
| Other clinics in hospitals | 20 (9.0) | (5.9, 13.6) | 33 (13.8) | (9.9, 18.7) | 7 (31.8) | | (15.4, 54.6) | 10 (45.5) | (25.6, 66.9) |
| CBOs | 3 (1.4) | (0.4, 4.1) | 14 (5.8) | (3.5, 9.6) | 17 (77.3) | | (54.3, 90.7) | 17 (77.3) | (54.3, 90.7) |
| Primary care centers | 13 (5.9) | (3.4, 9.9) | 22 (9.2) | (6.1, 13.6) | 19 (86.4) | | (63.7, 95.8) | 15 (68.2) | (45.4, 84.6) |
| Others | 3 (1.4) | (0.4, 4.1) | 8 (3.3) | (1.7, 6.5) | 3 (13.6) | | (4.2, 36.3) | 3 (13.6) | (4.2, 36.3) |
| **Psychosocial support providers (more than one answer possible)** |  |  |  |  |  | |  |  |  |
| Physicians | 182 (82.4) | (76.7, 86.9) | 177 (73.8) | (67.8, 79.0) | 18 (81.8) | | (59.0, 93.4) | 14 (63.6) | (41.2, 81.4) |
| Nurses | 72 (32.6) | (26.7, 39.1) | 55 (22.9) | (18.0, 28.7) | 17 (77.3) | | (54.3, 90.7) | 15 (68.2) | (45.4, 84.6) |
| Community health workers | 14 (6.3) | (3.8, 10.4) | 27 (11.3) | (7.8, 15.9) | 17 (77.3) | | (54.3, 90.7) | 17 (77.3) | (54.3, 90.7) |
| Health volunteers | 42 (19.0) | (14.3, 24.7) | 90 (37.5) | (31.6, 43.8) | 17 (77.3) | | (54.3, 90.7) | 19 (86.4) | (63.7, 95.8) |
| **Psychosocial support frequency** |  |  |  |  |  | |  |  |  |
| Monthly | 18 (8.3) | (5.3, 12.8) | 24 (10.1) | (6.8, 14.6) | 3 (13.6) | | (4.2, 36.3) | 4 (18.2) | (6.6, 41.0) |
| 2 months | 24 (11.0) | (7.5, 15.9) | 22 (9.2) | (6.2, 13.7) | 3 (13.6) | | (4.2, 36.3) | 3 (13.6) | (4.2, 36.3) |
| 3 months | 58 (26.6) | (21.1, 32.9) | 75 (31.5) | (25.9, 37.7) | 8 (36.4) | | (18.6, 58.8) | 8 (36.4) | (18.6, 58.8) |
| 6 months | 118 (54.1) | (47.4, 60.7) | 117 (49.2) | (42.8, 55.5) | 8 (36.4) | | (18.6, 58.8) | 7 (31.8) | (15.4, 54.6) |

95% CI, 95% confidence interval; ART, antiretroviral therapy; CBOs, community-based organizations; VL, viral load; STI, sexually transmitted infection.
