# Supplementary material for: Client and provider preferences for HIV care: Implications for implementing differentiated service delivery in Thailand
Source: J Int AIDS Soc. 2021 Mar 31;24(4):e25693. doi: 10.1002/jia2.25693 (PMC8013790; doi:10.1002/jia2.25693)
Supplement: Supplementary file 4 — Table S4. Service preferences by assigned sex at birth [file JIA2-24-e25693-s002.docx]

**S4 Table. Service preferences by assigned sex at birth.**

|  | **Clients** | | | | | **Providers** | | | |
| --- | --- | --- | --- | --- | --- | --- | --- | --- | --- |
|  | **Male**  **(N=263)**  **n (%)** | **95% CI** | **Female (N=234)**  **n (%)** | **95% CI** | **Male (N=10)**  **n (%)** | | **95% CI** | **Female (N=41)**  **n (%)** | **95% CI** |
| **ART refill locations (more than one answer possible)** |  |  |  |  |  | |  |  |  |
| ART clinics in hospitals | 222 (84.4) | (79.5, 88.3) | 201 (85.9) | (80.8, 89.8) | 9 (90.0) | | (49.1, 98.8) | 30 (73.2) | (57.1, 84.8) |
| Other clinics in hospitals | 26 (9.9) | (6.8, 14.1) | 29 (12.4) | (8.7, 17.3) | 2 (20.0) | | (4.5, 57.1) | 12 (29.3) | (17.1, 45.4) |
| CBOs | 16 (6.1) | (3.8, 9.7) | 17 (7.2) | (4.6, 11.4) | 5 (50.0) | | (20.8, 79.2) | 21 (51.2) | (35.7, 66.5) |
| Primary care centers | 31 (11.8) | (8.4, 16.3) | 18 (7.7) | (4.9, 11.9) | 7 (70.0) | | (35.1, 91.0) | 29 (70.7) | (54.6, 82.9) |
| Others | 10 (3.8) | (2.1, 6.9) | 10 (4.3) | (2.3, 7.8) | 0 | | - | 2 (4.9) | (1.2, 18.3) |
| **ART refill providers (more than one answer possible)** |  |  |  |  |  | |  |  |  |
| Physicians | 217 (82.5) | (77.4, 86.7) | 199 (85.0) | (79.9, 89.1) | 8 (80.0) | | (42.9, 95.5) | 33 (80.5) | (64.9, 90.2) |
| Nurses | 84 (31.9) | (26.6, 37.8) | 66 (28.2) | (22.8, 34.3) | 5 (50.0) | | (20.8, 79.2) | 29 (70.7) | (54.6, 82.9) |
| Community health workers | 22 (8.4) | (5.6, 12.4) | 21 (9.0) | (5.9, 13.4) | 2 (20.0) | | (4.5, 57.1) | 10 (24.4) | (13.3, 40.3) |
| Health volunteers | 37 (14.1) | (10.4, 18.8) | 35 (15.0) | (10.9, 20.1) | 0 | | - | 5 (12.2) | (5.0, 26.8) |
| **ART refill frequency** |  |  |  |  |  | |  |  |  |
| Monthly | 32 (12.2) | (8.8, 16.8) | 36 (15.5) | (11.4, 20.8) | 2 (20.0) | | (4.5, 57.1) | 3 (7.3) | (2.2, 21.1) |
| 2 months | 39 (14.9) | (11.1, 19.8) | 20 (8.6) | (5.6, 13.0) | 0 | | - | 1 (2.4) | (0.3, 16.4) |
| 3 months | 98 (37.4) | (31.7, 43.4) | 105 (45.3) | (38.9, 51.7) | 4 (40.0) | | (14.5, 72.3) | 24 (58.5) | (42.6, 72.9) |
| 6 months | 93 (35.5) | (29.9, 41.5) | 71 (30.6) | (25.0, 36.9) | 4 (40.0) | | (14.5, 72.3) | 13 (31.7) | (19.0, 47.9) |
| **VL testing locations (more than one answer possible)** |  |  |  |  |  | |  |  |  |
| ART clinics in hospitals | 239 (90.9) | (86.7, 93.8) | 212 (90.6) | (86.1, 93.7) | 10 (100) | | - | 33 (80.5) | (64.9, 90.2) |
| Other clinics in hospitals | 22 (8.4) | (5.6, 12.4) | 24 (10.3) | (7.0, 14.9) | 4 (40.0) | | (14.5, 72.3) | 17 (41.5) | (27.1, 57.4) |
| CBOs | 12 (4.6) | (2.6, 7.9) | 5 (2.1) | (0.9, 5.0) | 6 (60.0) | | (27.7, 85.5) | 18 (43.9) | (29.2, 59.7) |
| Primary care centers | 14 (5.3) | (3.2, 8.8) | 8 (3.4) | (1.7, 6.7) | 6 (60.0) | | (27.7, 85.5) | 22 (53.7) | (38.0, 68.6) |
| Others | 6 (2.3) | (1.0, 5.0) | 7 (3.0) | (1.4, 6.2) | 1 (10.0) | | (1.2, 50.9) | 3 (7.3) | (2.3, 21.1) |
| **VL testing providers (more than one answer possible)** |  |  |  |  |  | |  |  |  |
| Physicians | 248 (94.3) | (90.7, 96.5) | 222 (94.9) | (91.2, 97.1) | 10 (100) | | - | 41 (100) | - |
| Nurses | 49 (18.6) | (14.4, 23.8) | 38 (16.2) | (12.0, 21.6) | 5 (50.0) | | (20.8, 79.2) | 30 (73.2) | (57.1, 84.8) |
| Community health workers | 7 (2.7) | (1.3, 5.5) | 9 (3.8) | (2.0, 7.2) | 1 (10.0) | | (1.2, 50.9) | 11 (26.8) | (15.2, 42.9) |
| Health volunteers | 13 (4.9) | (2.9, 8.3) | 16 (6.8) | (4.2, 10.9) | 0 | | - | 8 (19.5) | (9.8, 35.1) |
| **VL testing frequency** |  |  |  |  |  | |  |  |  |
| Monthly | 19 (7.3) | (4.7, 11.2) | 26 (11.3) | (7.8, 16.1) | 0 | | - | 1 (2.4) | (0.3, 16.4) |
| 2 months | 24 (9.2) | (6.2, 13.4) | 10 (4.3) | (2.3, 7.9) | 0 | | - | 0 | - |
| 3 months | 65 (24.9) | (20.0, 30.5) | 67 (29.1) | (23.6, 35.4) | 1 (10.0) | | (1.2, 50.9) | 8 (19.5) | (9.8, 35.1) |
| 6 months | 153 (58.6) | (52.5, 64.5) | 127 (55.2) | (48.7, 61.6) | 9 (90.0) | | (49.1, 98.8) | 32 (78.0) | (62.3, 88.5) |
| **HIV/STI monitoring locations (more than one answer possible)** |  |  |  |  |  | |  |  |  |
| ART clinics in hospitals | 242 (92.0) | (88.0, 94.7) | 209 (89.3) | (84.6, 92.7) | 9 (90.0) | | (49.1, 98.8) | 34 (82.9) | (67.6, 91.9) |
| Other clinics in hospitals | 24 (9.1) | (6.2, 13.3) | 23 (9.8) | (6.6, 14.4) | 3 (30.0) | | (9.0, 64.9) | 17 (41.5) | (27.1, 57.4) |
| CBOs | 6 (2.3) | (1.0, 5.0) | 6 (2.6) | (1.2, 5.6) | 6 (60.0) | | (27.7, 85.5) | 23 (56.1) | (40.3, 70.8) |
| Primary care centers | 18 (6.8) | (4.3, 10.6) | 11 (4.7) | (2.6, 8.3) | 6 (60.0) | | (27.7, 85.5) | 30 (73.2) | (57.1, 84.8) |
| Others | 6 (2.3) | (1.0, 5.0) | 9 (3.8) | (2.0, 7.2) | 0 | | - | 2 (4.9) | (1.2, 18.3) |
| **HIV/STI monitoring providers (more than one answer possible)** |  |  |  |  |  | |  |  |  |
| Physicians | 232 (88.2) | (83.7, 91.6) | 205 (87.6) | (82.7, 91.3) | 6 (60.0) | | (27.7, 85.5) | 31 (75.6) | (59.7, 86.7) |
| Nurses | 73 (27.8) | (22.7, 33.5) | 54 (23.1) | (18.1, 28.9) | 9 (90.0) | | (49.1, 98.8) | 31 (75.6) | (59.7, 86.7) |
| Community health workers | 17 (6.5) | (4.0, 10.2) | 17 (7.3) | (4.6, 11.4) | 6 (60.0) | | (27.7, 85.5) | 28 (68.3) | (52.1, 81.0) |
| Health volunteers | 57 (21.7) | (17.1, 27.1) | 47 (20.1) | (15.4, 25.7) | 5 (50.0) | | (20.8, 79.2) | 24 (58.5) | (42.6, 72.9) |
| **HIV/STI monitoring frequency** |  |  |  |  |  | |  |  |  |
| Monthly | 25 (9.5) | (6.5, 13.8) | 34 (14.8) | (10.7, 20.0) | 1 (10.0) | | (1.2, 50.9) | 7 (17.1) | (8.1, 32.4) |
| 2 months | 29 (11.1) | (7.8, 15.5) | 15 (6.5) | (4.0, 10.6) | 3 (30.0) | | (9.0, 64.9) | 2 (4.9) | (1.2, 18.3) |
| 3 months | 84 (32.1) | (26.7, 38.0) | 86 (37.4) | (31.3, 43.9) | 2 (20.0) | | (4.5, 57.1) | 21 (51.2) | (35.7, 66.5) |
| 6 months | 124 (47.3) | (41.3, 53.5) | 95 (41.3) | (35.1, 47.8) | 4 (40.0) | | (14.5, 72.3) | 11 (26.8) | (15.2, 42.9) |
| **Psychosocial support locations (more than one answer possible)** |  |  |  |  |  | |  |  |  |
| ART clinics in hospitals | 229 (87.1) | (82.4, 90.6) | 195 (83.3) | (78.0, 87.6) | 10 (100) | | - | 33 (80.5) | (64.9, 90.2) |
| Other clinics in hospitals | 32 (12.2) | (8.7, 16.7) | 33 (14.1) | (10.2, 19.2) | 3 (30.0) | | (9.0, 64.9) | 18 (43.9) | (29.2, 59.7) |
| CBOs | 10 (3.8) | (2.1, 6.9) | 10 (4.3) | (2.3, 7.8) | 10 (100) | | - | 31 (75.6) | (59.7, 86.7) |
| Primary care centers | 23 (8.7) | (5.9, 12.8) | 17 (7.3) | (4.6, 11.4) | 9 (90.0) | | (49.1, 98.8) | 31 (75.6) | (59.7, 86.7) |
| Others | 7 (2.7) | (1.3, 5.5) | 8 (3.4) | (1.7, 6.7) | 3 (30.0) | | (9.0, 64.9) | 3 (7.3) | (2.3, 21.1) |
| **Psychosocial support providers (more than one answer possible)** |  |  |  |  |  | |  |  |  |
| Physicians | 201 (76.4) | (70.9, 81.2) | 181 (77.4) | (71.5, 82.3) | 7 (70.0) | | (35.1, 91.0) | 30 (73.2) | (57.1, 84.8) |
| Nurses | 81 (30.8) | (25.5, 36.7) | 58 (24.8) | (19.7, 30.7) | 9 (90.0) | | (49.1, 98.8) | 29 (70.7) | (54.6, 82.9) |
| Community health workers | 31 (11.8) | (8.4, 16.3) | 24 (10.3) | (7.0, 14.9) | 10 (100) | | - | 31 (24.4) | (13.3, 40.3) |
| Health volunteers | 83 (31.6) | (26.2, 37.5) | 72 (30.8) | (25.2, 37.0) | 10 (100) | | - | 33 (80.5) | (64.9, 90.2) |
| **Psychosocial support frequency** |  |  |  |  |  | |  |  |  |
| Monthly | 18 (6.9) | (4.4, 10.7) | 32 (13.9) | (10.0, 19.0) | 2 (20.0) | | (4.5, 57.1) | 5 (12.2) | (5.0, 26.8) |
| 2 months | 29 (11.1) | (7.8, 15.6) | 21 (9.1) | (6.0, 13.6) | 2 (20.0) | | (4.5, 57.1) | 7 (17.1) | (8.1, 32.4) |
| 3 months | 75 (28.7) | (23.5, 34.5) | 71 (30.7) | (25.1, 37.0) | 2 (20.0) | | (4.5, 57.1) | 16 (39.0) | (25.0, 55.1) |
| 6 months | 139 (53.3) | (47.2, 59.3) | 107 (46.3) | (40.0, 52.8) | 4 (40.0) | | (14.5, 72.3) | 13 (31.7) | (19.0, 47.9) |

95% CI, 95% confidence interval; ART, antiretroviral therapy; CBOs, community-based organizations; VL, viral load; STI, sexually transmitted infection.
