# Supplementary material for: Client and provider preferences for HIV care: Implications for implementing differentiated service delivery in Thailand
Source: J Int AIDS Soc. 2021 Mar 31;24(4):e25693. doi: 10.1002/jia2.25693 (PMC8013790; doi:10.1002/jia2.25693)
Supplement: Supplementary file 5 — Table S5. Service preferences by populations [file JIA2-24-e25693-s007.docx]

**S5 Table. Service preferences by populations.**

|  | **Clients** | | | | | | | | | | | | |
| --- | --- | --- | --- | --- | --- | --- | --- | --- | --- | --- | --- | --- | --- |
|  | **Gen.Pop. (N=395)**  **n (%)** | **95% CI** | | **MSM  (N=83)**  **n (%)** | | **95% CI** | **TGW  (N=12)**  **n (%)** | | **95% CI** | | **Other (N=5)**  **n (%)** | | **95% CI** |
| **ART refill locations (more than one answer possible)** |  |  | |  | |  |  | |  | |  | |  |
| ART clinics in hospitals | 337 (85.4) | (81.5, 88.5) | | 72 (86.7) | | (77.5, 92.5) | 10 (83.3) | | (50.5, 96.1) | | 3 (60.0) | | (16.8, 91.8) |
| Other clinics in hospitals | 45 (11.4) | (8.6, 14.9) | | 7 (8.4) | | (4.0, 16.7) | 2 (16.7) | | (3.9, 50.0) | | 1 (20.0) | | (2.1, 74.5) |
| CBOs | 28 (7.1) | (4.9, 10.1) | | 5 (6.0) | | (2.5, 13.8) | 0 | | - | | 0 | | - |
| Primary care centers | 36 (9.1) | (6.6, 12.4) | | 11 (13.3) | | (7.5, 22.5) | 1 (8.3) | | (1.1, 43.7) | | 0 | | - |
| Others | 16 (4.1) | (2.5, 6.5) | | 2 (2.4) | | (0.6, 9.2) | 1 (8.3) | | (1.1, 43.7) | | 1 (20.0) | | (2.1, 74.5) |
| **ART refill providers (more than one answer possible)** |  |  | |  | |  |  | |  | |  | |  |
| Physicians | 335 (84.8) | (80.9, 88.0) | | 66 (79.5) | | (69.4, 86.9) | 9 (75.0) | | (43.3, 92.2) | | 4 (80.0) | | (25.5, 97.9) |
| Nurses | 114 (28.9) | (24.6, 33.5) | | 28 (33.7) | | (24.3, 44.6) | 7 (58.3) | | (29.6, 82.3) | | 1 (20.0) | | (2.1, 74.5) |
| Community health workers | 34 (8.6) | (6.2, 11.8) | | 7 (8.4) | | (4.0, 16.7) | 2 (16.7) | | (3.9, 49.5) | | 0 | | - |
| Health volunteers | 59 (14.9) | (11.7, 18.8) | | 11 (13.3) | | (7.5, 22.5) | 2 (16.7) | | (3.9, 49.5) | | 0 | | - |
| **ART refill frequency** |  |  | |  | |  |  | |  | |  | |  |
| Monthly | 58 (14.8) | (11.6, 18.6) | | 7 (8.5) | | (4.1, 16.9) | 1 (8.3) | | (1.1, 43.7) | | 1 (20.0) | | (2.1, 74.5) |
| 2 months | 42 (10.7) | (8.0, 14.2) | | 17 (20.7) | | (13.2, 30.9) | 0 | | - | | 1 (20.0) | | (2.1, 74.5) |
| 3 months | 158 (40.2) | (35.4, 45.2) | | 37 (45.1) | | (34.6, 56.0) | 7 (58.3) | | (29.6, 82.3) | | 0 | | - |
| 6 months | 135 (34.4) | (29.8, 39.2) | | 21 (25.6) | | (17.3, 36.2) | 4 (33.3) | | (12.5, 63.7) | | 3 (60.0) | | (16.8, 91.8) |
| **VL testing locations (more than one answer possible)** |  |  | |  | |  |  | |  | |  | |  |
| ART clinics in hospitals | 356 (90.1) | (86.8, 92.7) | | 78 (94.0) | | (86.2, 97.5) | 11 (91.7) | | (56.3, 98.9) | | 4 (80.0) | | (25.5, 97.9) |
| Other clinics in hospitals | 38 (9.6) | (7.1, 13.0) | | 6 (7.2) | | (3.3, 15.3) | 1 (8.3) | | (1.1, 43.7) | | 1 (20.0) | | (2.1, 74.5) |
| CBOs | 13 (3.3) | (1.9, 5.6) | | 4 (4.8) | | (1.8, 12.2) | 0 | | - | | 0 | | - |
| Primary care centers | 15 (3.8) | (2.3, 6.2) | | 6 (7.2) | | (3.3, 15.3) | 1 (8.3) | | (1.1, 43.7) | | 0 | | - |
| Others | 12 (3.0) | (1.7, 5.3) | | 1 (1.2) | | (0.2, 8.2) | 0 | | - | | 0 | | - |
| **VL testing providers (more than one answer possible)** |  |  | |  | |  |  | |  | |  | |  |
| Physicians | 372 (94.2) | (91.4, 96.1) | | 79 (95.2) | | (87.8, 98.2) | 12 (100) | | - | | 5 (100) | | - |
| Nurses | 68 (17.2) | (13.8, 21.3) | | 17 (20.5) | | (13.1, 30.6) | 2 (16.7) | | (3.9, 49.5) | | 0 | | - |
| Community health workers | 14 (3.5) | (2.1, 5.9) | | 2 (2.4) | | (0.6, 9.2) | 0 | | - | | 0 | | - |
| Health volunteers | 27 (6.8) | (4.7, 9.8) | | 2 (2.4) | | (0.6, 9.2) | 0 | | - | | 0 | | - |
| **VL testing frequency** |  |  | |  | |  |  | |  | |  | |  |
| Monthly | 37 (9.5) | (6.9, 12.8) | | 6 (7.3) | | (3.3, 15.4) | 0 | | - | | 1 (20.0) | | (2.1, 74.5) |
| 2 months | 22 (5.6) | (3.7, 8.4) | | 10 (12.2) | | (6.7, 21.3) | 2 (16.7) | | (3.9, 49.5) | | 1 (20.0) | | (2.1, 74.5) |
| 3 months | 107 (27.4) | (23.2, 32.1) | | 23 (28.0) | | (19.3, 38.8) | 2 (16.7) | | (3.9, 49.5) | | 0 | | - |
| 6 months | 224 (57.4) | (52.5, 62.3) | | 43 (52.4) | | (41.6, 63.1) | 8 (66.7) | | (36.3, 87.5) | | 3 (60.0) | | (16.8, 91.8) |
| **HIV/STI monitoring locations (more than one answer possible)** |  |  | |  | |  |  | |  | |  | |  |
| ART clinics in hospitals | 355 (89.9) | (86.5, 92.5) | | 80 (96.4) | | (89.3, 98.8) | 11 (91.7) | | (56.3, 98.9) | | 3 (60.0) | | (16.8, 91.8) |
| Other clinics in hospitals | 38 (9.6) | (7.1, 13.0) | | 6 (7.2) | | (3.3, 15.3) | 2 (16.7) | | (3.9, 49.5) | | 1 (20.0) | | (2.1, 74.5) |
| CBOs | 11 (2.8) | (1.5, 5.0) | | 1 (1.2) | | (0.2, 8.2) | 0 | | - | | 0 | | - |
| Primary care centers | 21 (5.3) | (3.5, 8.0) | | 8 (9.6) | | (4.9, 18.2) | 0 | | - | | 0 | | - |
| Others | 13 (3.3) | (1.9, 5.6) | | 1 (1.2) | | (0.2, 8.2) | 0 | | - | | 1 (20.0) | | (2.1, 74.5) |
| **HIV/STI monitoring providers (more than one answer possible)** |  |  | |  | |  |  | |  | |  | |  |
| Physicians | 348 (88.1) | (84.5, 91.0) | | 76 (91.6) | | (83.3, 96.0) | 9 (75.0) | | (43.3, 92.2) | | 3 (60.0) | | (16.8, 91.8) |
| Nurses | 94 (23.8) | (19.8, 28.3) | | 26 (31.3) | | (22.2, 42.1) | 6 (50.0) | | (23.4, 76.6) | | 1 (20.0) | | (2.1, 74.5) |
| Community health workers | 29 (7.3) | (5.1, 10.4) | | 4 (4.8) | | (1.8, 12.2) | 1 (8.3) | | (1.1, 43.7) | | 0 | | - |
| Health volunteers | 83 (21.0) | (17.3, 25.3) | | 13 (15.7) | | (9.3, 25.2) | 6 (50.0) | | (23.4, 76.6) | | 1 (20.0) | | (2.1, 74.5) |
| **HIV/STI monitoring frequency** |  |  | |  | |  |  | |  | |  | |  |
| Monthly | 50 (12.8) | (9.8, 16.5) | | 6 (7.2) | | (3.3, 15.3) | 1 (8.3) | | (1.1, 43.7) | | 1 (20.0) | | (2.1, 74.5) |
| 2 months | 28 (7.2) | (5.0, 10.2) | | 15 (18.1) | | (11.2, 27.9) | 1 (8.3) | | (1.1, 43.7) | | 1 (20.0) | | (2.1, 74.5) |
| 3 months | 132 (33.8) | (29.3, 38.7) | | 31 (37.3) | | (27.6, 48.3) | 6 (50.0) | | (23.4, 76.6) | | 0 | | - |
| 6 months | 180 (46.2) | (41.2, 51.1) | | 31 (37.3) | | (27.6, 48.3) | 4 (33.3) | | (12.5, 63.7) | | 3 (60.0) | | (16.8, 91.8) |
| **Psychosocial support locations (more than one answer possible)** |  |  | |  | |  |  | |  | |  | |  |
| ART clinics in hospitals | 332 (84.1) | (80.1, 87.4) | | 78 (94.0) | | (86.2, 97.5) | 10 (83.3) | | (50.5, 96.1) | | 2 (40.0) | | (8.2, 83.2) |
| Other clinics in hospitals | 54 (13.7) | (10.6, 17.4) | | 7 (8.4) | | (4.0, 16.7) | 3 (25.0) | | (7.8, 56.7) | | 1 (20.0) | | (2.1, 74.5) |
| CBOs | 19 (4.8) | (3.1, 7.4) | | 1 (1.2) | | (0.2, 8.2) | 0 | | - | | 0 | | - |
| Primary care centers | 30 (7.6) | (5.4, 10.7) | | 8 (9.6) | | (4.9, 18.2) | 1 (8.3) | | (1.1, 43.7) | | 1 (20.0) | | (2.1, 74.5) |
| Others | 14 (3.5) | (2.1, 5.9) | | 1 (1.2) | | (0.2, 8.2) | 0 | | - | | 0 | | - |
| **Psychosocial support providers (more than one answer possible)** |  |  | |  | |  |  | |  | |  | |  |
| Physicians | 304 (77.0) | (72.5, 80.7) | | 65 (78.3) | | (68.1, 85.9) | 10 (83.3) | | (50.5, 96.1) | | 2 (40.0) | | (8.2, 83.2) |
| Nurses | 105 (26.6) | (22.4, 31.2) | | 27 (32.5) | | (23.3, 43.4) | 6 (50.0) | | (23.4, 76.6) | | 1 (20.0) | | (2.1, 74.5) |
| Community health workers | 43 (10.9) | (8.2, 14.4) | | 10 (12.0) | | (6.6, 21.1) | 2 (16.7) | | (3.9, 49.5) | | 0 | | - |
| Health volunteers | 125 (31.6) | (27.2, 36.4) | | 22 (26.5) | | (18.1, 37.1) | 5 (41.7) | | (17.7, 70.4) | | 2 (40.0) | | (8.2, 83.2) |
| **Psychosocial support frequency** |  |  | |  | |  |  | |  | |  | |  |
| Monthly | 43 (10.8) | (8.0, 14.3) | | 5 (6.0) | | (2.5, 13.8) | 1 (8.3) | | (1.1, 43.7) | | 1 (20.0) | | (2.1, 74.5) |
| 2 months | 35 (9.0) | (6.5, 12.3) | | 13 (15.7) | | (9.3, 25.2) | 1 (8.3) | | (1.1, 43.7) | | 2 (40.0) | | (8.2, 83.2) |
| 3 months | 116 (29.7) | (25.4, 34.5) | | 25 (30.1) | | (21.2, 40.9) | 4 (33.3) | | (12.5, 63.7) | | 0 | | - |
| 6 months | 197 (50.5) | (45.5, 55.5) | | 40 (48.2) | | (37.6, 59.0) | 6 (50.0) | | (23.4, 76.6) | | 2 (40.0) | | (8.2, 83.2) |
|  | **Providers** | | | | | | | | | | | | |
|  | **Gen.Pop.  (N= 47)**  **n (%)** | | **95% CI** | | **MSM (N=1)**  **n (%)** | | | **95% CI** | | **TGW (N=4)**  **n (%)** | | **95% CI** | |
| **ART refill locations (more than one answer possible)** |  | |  | |  | | |  | |  | |  | |
| ART clinics in hospitals | 36 (76.6) | | (61.9, 86.8) | | 1 (100) | | | - | | 3 (75.0) | | (17.1, 97.8) | |
| Other clinics in hospitals | 13 (27.7) | | (16.5, 42.6) | | 1 (100) | | | - | | 1 (25.0) | | (2.2, 82.9) | |
| CBOs | 23 (48.9) | | (34.6, 63.4) | | 1 (100) | | | - | | 2 (50.0) | | (9.0, 91.0) | |
| Primary care centers | 32 (68.1) | | (53.1, 80.1) | | 1 (100) | | | - | | 4 (100) | | - | |
| Others | 2 (4.3) | | (1.0, 16.2) | | 0 | | | - | | 0 | | - | |
| **ART refill providers (more than one answer possible)** |  | |  | |  | | |  | |  | |  | |
| Physicians | 38 (80.9) | | (66.6, 90.0) | | 1 (100) | | | - | | 3 (75.0) | | (17.1, 97.8) | |
| Nurses | 32 (68.1) | | (53.1, 80.1) | | 0 | | | - | | 2 (50.0) | | (9.0, 91.0) | |
| Community health workers | 10 (21.3) | | (11.6, 35.8) | | 0 | | | - | | 2 (50.0) | | (9.0, 91.0) | |
| Health volunteers | 5 (10.6) | | (4.4, 23.7) | | 0 | | | - | | 0 | | - | |
| **ART refill frequency** |  | |  | |  | | |  | |  | |  | |
| Monthly | 4 (8.5) | | (3.1, 21.2) | | 0 | | | - | | 1 (25.0) | | (2.2, 82.9) | |
| 2 months | 1 (2.1) | | (0.3, 14.5) | | 0 | | | - | | 0 | | - | |
| 3 months | 27 (57.4) | | (42.6, 71.1) | | 0 | | | - | | 2 (50.0) | | (9.0, 91.0) | |
| 6 months | 15 (31.9) | | (19.9, 46.9) | | 1 (100) | | | - | | 1 (25.0) | | (2.2, 82.9) | |
| **VL testing locations (more than one answer possible)** |  | |  | |  | | |  | |  | |  | |
| ART clinics in hospitals | 39 (83.0) | | (68.9, 91.5) | | 1 (100) | | | - | | 4 (100) | | - | |
| Other clinics in hospitals | 18 (38.3) | | (25.2, 53.3) | | 1 (100) | | | - | | 2 (50.0) | | (9.0, 91.0) | |
| CBOs | 19 (40.4) | | (27.1, 55.4) | | 1 (100) | | | - | | 4 (100) | | - | |
| Primary care centers | 24 (51.1) | | (36.6, 65.3) | | 1 (100) | | | - | | 3 (75.0) | | (17.1, 97.8) | |
| Others | 4 (8.5) | | (3.1, 21.2) | | 0 | | | - | | 0 | | - | |
| **VL testing providers (more than one answer possible)** |  | |  | |  | | |  | |  | |  | |
| Physicians | 47 (100) | | - | | 1 (100) | | | - | | 4 (100) | | - | |
| Nurses | 32 (68.1) | | (53.1, 80.1) | | 1 (100) | | | - | | 2 (50.0) | | (9.0, 91.0) | |
| Community health workers | 11 (23.4) | | (13.2, 38.1) | | 0 | | | - | | 1 (25.0) | | (2.2, 82.9) | |
| Health volunteers | 8 (17.0) | | (8.5, 31.1) | | 0 | | | - | | 0 | | - | |
| **VL testing frequency** |  | |  | |  | | |  | |  | |  | |
| Monthly | 1 (2.1) | | (0.3, 14.5) | | 0 | | | - | | 0 | | - | |
| 2 months | 0 | | - | | 0 | | | - | | 0 | | - | |
| 3 months | 8 (17.0) | | (8.5, 31.1) | | 0 | | | - | | 1 (25.0) | | (2.2, 82.9) | |
| 6 months | 38 (80.9) | | (66.6, 90.0) | | 1 (100) | | | - | | 3 (75.0) | | (17.1, 97.8) | |
| **HIV/STI monitoring locations (more than one answer possible)** |  | |  | |  | | |  | |  | |  | |
| ART clinics in hospitals | 39 (83.0) | | (68.9, 91.5) | | 1 (100) | | | - | | 4 (100) | | - | |
| Other clinics in hospitals | 17 (36.2) | | (23.4, 51.2) | | 1 (100) | | | - | | 2 (50.0) | | (9.0, 91.0) | |
| CBOs | 25 (53.2) | | (38.6, 67.3) | | 1 (100) | | | - | | 3 (75.0) | | (17.1, 97.8) | |
| Primary care centers | 32 (68.1) | | (53.1, 80.1) | | 1 (100) | | | - | | 3 (75.0) | | (17.1, 97.8) | |
| Others | 2 (4.3) | | (1.0, 16.2) | | 0 | | | - | | 0 | | - | |
| **HIV/STI monitoring providers (more than one answer possible)** |  | |  | |  | | |  | |  | |  | |
| Physicians | 34 (72.3) | | (57.4, 83.5) | | 1 (100) | | | - | | 2 (50.0) | | (9.0, 91.0) | |
| Nurses | 38 (80.9) | | (66.6, 90.0) | | 1 (100) | | | - | | 2 (50.0) | | (9.0, 91.0) | |
| Community health workers | 31 (66.0) | | (50.9, 78.3) | | 1 (100) | | | - | | 3 (75.0) | | (17.1, 97.8) | |
| Health volunteers | 27 (57.4) | | (42.6, 71.1) | | 1 (100) | | | - | | 2 (50.0) | | (9.0, 91.0) | |
| **HIV/STI monitoring frequency** |  | |  | |  | | |  | |  | |  | |
| Monthly | 8 (17.0) | | (8.5, 31.1) | | 0 | | | - | | 0 | | - | |
| 2 months | 3 (6.4) | | (2.0, 18.6) | | 0 | | | - | | 2 (50.0) | | (9.0, 91.0) | |
| 3 months | 23 (48.9) | | (34.6, 63.4) | | 0 | | | - | | 1 (25.0) | | (2.2, 82.9) | |
| 6 months | 13 (27.7) | | (16.5, 42.6) | | 1 (100) | | | - | | 1 (25.0) | | (2.2, 82.9) | |
| **Psychosocial support locations (more than one answer possible)** |  | |  | |  | | |  | |  | |  | |
| ART clinics in hospitals | 40 (85.1) | | (71.3, 92.9) | | 1 (100) | | | - | | 3 (75.0) | | (17.1, 97.8) | |
| Other clinics in hospitals | 19 (40.4) | | (27.1, 55.4) | | 1 (100) | | | - | | 1 (25.0) | | (2.2, 82.9) | |
| CBOs | 37 (78.7) | | (64.2, 88.4) | | 1 (100) | | | - | | 4 (100) | | - | |
| Primary care centers | 35 (74.5) | | (60.0, 85.2) | | 1 (100) | | | - | | 4 (100) | | - | |
| Others | 6 (12.8) | | (5.7, 26.2) | | 0 | | | - | | 0 | | - | |
| **Psychosocial support providers (more than one answer possible)** |  | |  | |  | | |  | |  | |  | |
| Physicians | 35 (74.5) | | (60.0, 85.2) | | 1 (100) | | | - | | 1 (25.0) | | (2.2, 82.9) | |
| Nurses | 34 (72.3) | | (57.4, 83.5) | | 1 (100) | | | - | | 3 (75.0) | | (17.1, 97.8) | |
| Community health workers | 37 (78.7) | | (64.2, 88.4) | | 1 (100) | | | - | | 4 (100) | | - | |
| Health volunteers | 39 (83.0) | | (68.9, 91.5) | | 1 (100) | | | - | | 4 (100) | | - | |
| **Psychosocial support frequency** |  | |  | |  | | |  | |  | |  | |
| Monthly | 7 (14.9) | | (7.1, 28.7) | | 0 | | | - | | 1 (25.0) | | (2.2, 82.9) | |
| 2 months | 8 (17.0) | | (8.5, 31.1) | | 0 | | | - | | 1 (25.0) | | (2.2, 82.9) | |
| 3 months | 17 (36.2) | | (23.4, 51.2) | | 0 | | | - | | 1 (25.0) | | (2.2, 82.9) | |
| 6 months | 15 (31.9) | | (19.9, 46.9) | | 1 (100) | | | - | | 1 (25.0) | | (2.2, 82.9) | |

Gen. Pop., general population; MSM, men who have sex with men; TGW, transgender women; 95% CI, 95% confidence interval; ART, antiretroviral therapy; CBOs, community-based organizations; VL, viral load; STI, sexually transmitted infection.
