# Supplementary material for: Client and provider preferences for HIV care: Implications for implementing differentiated service delivery in Thailand
Source: J Int AIDS Soc. 2021 Mar 31;24(4):e25693. doi: 10.1002/jia2.25693 (PMC8013790; doi:10.1002/jia2.25693)
Supplement: Supplementary file 6 — Table S6. Service preferences by study sites [file JIA2-24-e25693-s004.docx]

**S6 Table. Service preferences by study sites.**

|  | **Clients** | | | | | | | | | | | | | | | | | | |
| --- | --- | --- | --- | --- | --- | --- | --- | --- | --- | --- | --- | --- | --- | --- | --- | --- | --- | --- | --- |
|  | **TRC (N=100)**  **n (%)** | **95% CI** | | **QSV  (N=100)**  **n (%)** | | **95% CI** | | **NKP  (N=100)**  **n (%)** | | **95% CI** | | **SRP  (N=100)**  **n (%)** | | **95% CI** | | **HY  (N=100)**  **n (%)** | | **95% CI** | |
| **ART refill locations (more than one answer possible)** |  | |  | |  | |  | |  | |  | |  | |  | |  | |  |
| **ART clinics in hospitals** | 85 (85.0) | | (76.5, 90.8) | | 85 (85.0) | | (76.5, 90.8) | | 91 (91.0) | | (83.5, 95.3) | | 81 (81.0) | | (72.0, 87.6) | | 84 (84.0) | | (75.4, 90.0) |
| **Other clinics in hospitals** | 8 (8.0) | | (4.0, 15.3) | | 8 (8.0) | | (4.0, 15.3) | | 9 (9.0) | | (4.7, 16.5) | | 16 (16.0) | | (10.0, 24.6) | | 14 (14.0) | | (8.4, 22.3) |
| **CBOs** | 6 (6.0) | | (2.7, 12.8) | | 6 (6.0) | | (2.7, 12.8) | | 2 (2.0) | | (0.5, 7.7) | | 10 (10.0) | | (5.4, 17.7) | | 9 (9.0) | | (4.7, 16.5) |
| **Primary care centers** | 15 (15.0) | | (9.2, 23.5) | | 8 (8.0) | | (4.0, 15.3) | | 10 (10.0) | | (5.4, 17.7) | | 10 (10.0) | | (5.4, 17.7) | | 6 (6.0) | | (2.7, 12.8) |
| **Others** | 2 (2.0) | | (0.5, 7.7) | | 5 (5.0) | | (2.1, 11.5) | | 2 (2.0) | | (0.5, 7.7) | | 7 (7.0) | | (3.4, 14.0) | | 4 (4.0) | | (1.5, 10.2) |
| **ART refill providers (more than one answer possible)** |  | |  | |  | |  | |  | |  | |  | |  | |  | |  |
| **Physicians** | 78 (78.0) | | (68.8, 85.1) | | 87 (87.0) | | (78.8, 92.3) | | 82 (82.0) | | (73.2, 88.4) | | 87 (87.0) | | (78.8, 92.3) | | 85 (85.0) | | (76.5, 90.8) |
| **Nurses** | 36 (36.0) | | (27.2, 45.9) | | 19 (19.0) | | (12.4, 28.0) | | 31 (31.0) | | (22.7, 40.8) | | 36 (36.0) | | (27.2, 45.9) | | 28 (28.0) | | (20.0, 37.6) |
| **Community health workers** | 5 (5.0) | | (2.1, 11.5) | | 7 (7.0) | | (3.4, 14.0) | | 7 (7.0) | | (3.4, 14.0) | | 16 (16.0) | | (10.0, 24.6) | | 8 (8.0) | | (4.0, 15.3) |
| **Health volunteers** | 10 (10.0) | | (5.4, 17.7) | | 14 (14.0) | | (8.4, 22.3) | | 13 (13.0) | | (7.7, 21.2) | | 22 (22.0) | | (14.9, 31.2) | | 13 (13.0) | | (7.7, 21.2) |
| **ART refill frequency** |  | |  | |  | |  | |  | |  | |  | |  | |  | |  |
| **Monthly** | 7 (7.1) | | (3.4, 14.2) | | 14 (14.0) | | (8.4, 22.3) | | 11 (11.2) | | (6.3, 19.2) | | 15 (15.0) | | (9.2, 23.5) | | 22 (22.0) | | (14.9, 31.2) |
| **2 months** | 24 (24.2) | | (16.8, 33.7) | | 9 (9.0) | | (4.7, 16.5) | | 14 (14.3) | | (8.6, 22.8) | | 11 (11.0) | | (6.2, 18.9) | | 3 (3.0) | | (1.0,  9.0) |
| **3 months** | 35 (35.4) | | (26.5, 45.3) | | 41 (41.0) | | (31.7, 50.9) | | 36 (36.7) | | (27.7, 46.8) | | 50 (50.0) | | (40.3, 59.7) | | 41 (41.0) | | (31.7, 50.9) |
| **6 months** | 33 (33.3) | | (24.7, 43.2) | | 36 (36.0) | | (27.2, 45.9) | | 37 (37.8) | | (28.7, 47.8) | | 24 (24.0) | | (16.6, 33.4) | | 34 (34.0) | | (25.3, 43.9) |
| **VL testing locations (more than one answer possible)** |  | |  | |  | |  | |  | |  | |  | |  | |  | |  |
| **ART clinics in hospitals** | 94 (94.0) | | (87.2, 97.3) | | 93 (93.0) | | (86.0, 96.6) | | 94 (94.0) | | (87.2, 97.3) | | 81 (81.0) | | 72.0, 87.6) | | 92 (92.0) | | (84.7, 96.0) |
| **Other clinics in hospitals** | 4 (4.0) | | (1.5, 10.2) | | 3 (3.0) | | (1.0, 9.0) | | 7 (7.0) | | (3.4, 14.0) | | 21 (21.0) | | (14.1, 30.2) | | 11 (11.0) | | (6.2, 18.9) |
| **CBOs** | 4 (4.0) | | (1.5, 10.2) | | 2 (2.0) | | (0.5, 10.2) | | 2 (2.0) | | (0.5, 10.2) | | 4 (4.0) | | (1.5, 10.2) | | 5 (5.0) | | (2.1, 11.5) |
| **Primary care centers** | 7 (7.0) | | (3.4, 14.0) | | 1 (1.0) | | (0.1, 6.8) | | 5 (5.0) | | (2.1, 11.5) | | 6 (6.0) | | (2.7, 12.8) | | 3 (3.0) | | (1.0, 9.0) |
| **Others** | 1 (1.0) | | (0.1, 6.8) | | 2 (2.0) | | (0.5, 10.2) | | 1 (1.0) | | (0.1, 6.8) | | 5 (5.0) | | (2.1, 11.5) | | 4 (4.0) | | (1.5, 10.2) |
| **VL testing providers (more than one answer possible)** |  | |  | |  | |  | |  | |  | |  | |  | |  | |  |
| **Physicians** | 94 (94.0) | | (87.2, 97.3) | | 94 (94.0) | | (87.2, 97.3) | | 93 (93.0) | | (86.0, 96.6) | | 96 (96.0) | | (89.8, 98.5) | | 96 (96.0) | | (89.8, 98.5) |
| **Nurses** | 19 (19.0) | | (12.4, 28.0) | | 19 (19.0) | | (12.4, 28.0) | | 13 (13.0) | | (7.7, 21.2) | | 25 (25.0) | | (17.4, 34.5) | | 11 (11.0) | | (6.2, 18.9) |
| **Community health workers** | 1 (1.0) | | (0.1, 6.8) | | 3 (3.0) | | (1.0, 9.0) | | 0 | | - | | 9 (9.0) | | (4.7, 16.5) | | 3 (3.0) | | (1.0, 9.0) |
| **Health volunteers** | 2 (2.0) | | (0.5, 10.2) | | 5 (5.0) | | (2.1, 11.5) | | 5 (5.0) | | (2.1, 11.5) | | 12 (12.0) | | (6.9, 20.0) | | 5 (5.0) | | (2.1, 11.5) |
| **VL testing frequency** |  | |  | |  | |  | |  | |  | |  | |  | |  | |  |
| **Monthly** | 7 (7.1) | | (3.4, 14.2) | | 10 (10.0) | | (5.4, 17.7) | | 4 (4.2) | | (1.6, 10.8) | | 16 (16.0) | | (10.0, 24.6) | | 9 (9.00 | | (4.7, 16.5) |
| **2 months** | 14 (14.1) | | (8.5, 22.5) | | 6 (6.0) | | (2.7, 12.8) | | 5 (5.3) | | (2.2, 12.1) | | 10 (10.0) | | (5.4, 17.7) | | 1 (1.0) | | (0.1, 6.8) |
| **3 months** | 22 (22.2) | | (15.1, 31.5) | | 26 (26.0) | | (18.3, 35.5) | | 27 (28.4) | | (20.2, 38.4) | | 30 (30.0) | | (21.8, 39.7) | | 27 (27.0) | | (19.2, 36.6) |
| **6 months** | 56 (56.6) | | (46.6, 66.0) | | 58 (58.0) | | (48.1, 67.3) | | 59 (62.1) | | (51.9, 71.3) | | 44 (44.0) | | (34.5, 53.9) | | 63 (63.0) | | (53.1, 71.9) |
| **HIV/STI monitoring locations (more than one answer possible)** |  | |  | |  | |  | |  | |  | |  | |  | |  | |  |
| **ART clinics in hospitals** | 95 (95.0) | | (88.5, 97.9) | | 90 (90.0) | | (82.3, 94.6) | | 93 (93.0) | | (86.0, 96.6) | | 86 (86.0) | | (77.7, 91.6) | | 90 (90.0) | | (82.3, 94.6) |
| **Other clinics in hospitals** | 6 (6.0) | | (2.7, 12.8) | | 5 (5.0) | | (2.1, 11.5) | | 6 (6.0) | | (2.7, 12.8) | | 17 (17.0) | | (10.8, 25.7) | | 13 (13.0) | | (7.7, 21.2) |
| **CBOs** | 3 (3.0) | | (1.0, 9.0) | | 2 (2.0) | | (0.5, 10.2) | | 2 (2.0) | | (0.5, 10.2) | | 4 (4.0) | | (1.5, 10.2) | | 1 (1.0) | | (0.1, 6.8) |
| **Primary care centers** | 6 (6.0) | | (2.7, 12.8) | | 4 (4.0) | | (1.5, 10.2) | | 4 (4.0) | | (1.5, 10.2) | | 11 (11.0) | | (6.2, 10.2) | | 4 (4.0) | | (1.5, 10.2) |
| **Others** | 1 (1.0) | | (0.1, 6.8) | | 3 (3.0) | | (1.0, 9.0) | | 1 (1.0) | | (0.1, 6.8) | | 4 (4.0) | | (1.5, 10.2) | | 6 (6.0) | | (2.7, 12.8) |
| **HIV/STI monitoring providers (more than one answer possible)** |  | |  | |  | |  | |  | |  | |  | |  | |  | |  |
| **Physicians** | 87 (87.0) | | (78.8, 92.3) | | 88 (88.0) | | (80.0, 93.1) | | 91 (91.0) | | (83.5, 95.3) | | 88 (88.0) | | (80.0, 93.1) | | 86 (86.0) | | (77.7, 91.6) |
| **Nurses** | 27 (27.0) | | (19.2, 36.6) | | 23 (23.0) | | (15.7, 32.3) | | 26 (26.0) | | (18.3, 35.5) | | 30 (30.0) | | (21.8, 39.7) | | 21 (21.0) | | (14.1, 30.2) |
| **Community health workers** | 2 (2.0) | | (0.5, 10.2) | | 8 (8.0) | | (4.0, 15.3) | | 7 (7.0) | | (3.4, 14.0) | | 11 (11.0) | | (6.2, 10.2) | | 6 (6.0) | | (2.7, 12.8) |
| **Health volunteers** | 13 (13.0) | | (7.7, 21.2) | | 22 (22.0) | | (14.9, 31.2) | | 13 (13.0) | | (7.7, 21.2) | | 36 (36.0) | | (27.2, 45.9) | | 20 (20.0) | | (13.2, 29.1) |
| **HIV/STI monitoring frequency** |  | |  | |  | |  | |  | |  | |  | |  | |  | |  |
| **Monthly** | 10 (10.1) | | (5.5, 17.8) | | 13 (13.1) | | (7.7, 21.4) | | 9 (9.3) | | (4.9, 17.0) | | 18 (18.0) | | (11.6, 26.8) | | 10 (10.0) | | (5.4, 17.7) |
| **2 months** | 17 (17.2) | | (10.9, 26.0) | | 9 (9.1) | | (4.8, 16.6) | | 9 (9.3) | | (4.9, 17.0) | | 9 (9.0) | | (4.7, 16.5) | | 2 (2.0) | | (0.5,  7.7) |
| **3 months** | 28 (28.3) | | (20.2, 38.0) | | 25 (25.3) | | (17.6, 34.8) | | 36 (37.1) | | (28.0, 47.2) | | 43 (43.0) | | (33.6, 52.9) | | 38 (38.0) | | (29.0, 47.9) |
| **6 months** | 44 (44.4) | | (34.9, 54.4) | | 52 (52.5) | | (42.6, 62.2) | | 43 (44.3) | | (34.7, 54.4) | | 30 (30.0) | | (21.8, 39.7) | | 50 (50.0) | | (40.3, 59.7) |
| **Psychosocial support locations (more than one answer possible)** |  | |  | |  | |  | |  | |  | |  | |  | |  | |  |
| **ART clinics in hospitals** | 92 (92.0) | | (84.7, 96.0) | | 79 (79.0) | | (69.8, 85.9) | | 90 (90.0) | | (82.3, 94.6) | | 81 (81.0) | | (72.0, 87.6) | | 85 (85.0) | | (76.5, 90.8) |
| **Other clinics in hospitals** | 7 (7.0) | | (3.4, 14.0) | | 13 (13.0) | | (7.7, 21.2) | | 10 (10.0) | | (5.4, 17.7) | | 21 (21.0) | | (14.1, 30.2) | | 14 (14.0) | | (8.4, 22.3) |
| **CBOs** | 3 (3.0) | | (1.0, 9.0) | | 6 (6.0) | | (2.7, 12.8) | | 1 (1.0) | | (0.1, 6.8) | | 9 (9.0) | | (4.7, 16.5) | | 1 (1.0) | | (0.1, 6.8) |
| **Primary care centers** | 9 (9.0) | | (4.7, 16.5) | | 7 (7.0) | | (3.4, 14.0) | | 6 (6.0) | | (2.7, 12.8) | | 10 (10.0) | | (5.4, 17.7) | | 8 (8.0) | | (4.0, 15.3) |
| **Others** | 1 (1.0) | | (0.1, 6.8) | | 4 (4.0) | | (1.5, 10.2) | | 1 (1.0) | | (0.1, 6.8) | | 4 (4.0) | | (1.5, 10.2) | | 5 (5.0) | | (2.1, 11.5) |
| **Psychosocial support providers (more than one answer possible)** |  | |  | |  | |  | |  | |  | |  | |  | |  | |  |
| **Physicians** | 77 (77.0) | | (67.7, 84.3) | | 76 (76.0) | | (66.6, 83.4) | | 83 (83.0) | | (74.3, 89.2) | | 77 (77.0) | | (67.7, 84.3) | | 72 (72.0) | | (62.4, 80.0) |
| **Nurses** | 30 (30.0) | | (21.8, 39.7) | | 25 (25.0) | | (17.4, 34.5) | | 23 (23.0) | | (15.7, 32.3) | | 33 (33.0) | | (24.5, 42.8) | | 28 (28.0) | | (20.0, 37.6) |
| **Community health workers** | 6 (6.0) | | (2.7, 12.8) | | 10 (10.0) | | (5.4, 17.7) | | 8 (8.0) | | (4.0, 15.3) | | 21 (21.0) | | (14.1, 30.2) | | 10 (10.0) | | (5.4, 17.7) |
| **Health volunteers** | 24 (24.0) | | (16.6, 33.4) | | 27 (27.0) | | (19.2, 36.6) | | 24 (24.0) | | (16.6, 33.4) | | 45 (45.0) | | (35.5, 54.9) | | 35 (35.0) | | (26.2, 44.9) |
| **Psychosocial support frequency** |  | |  | |  | |  | |  | |  | |  | |  | |  | |  |
| **Monthly** | 6 (6.0) | | (2.7, 12.8) | | 13 (13.0) | | (7.7, 21.2) | | 5 (5.2) | | (2.2, 12.0) | | 15 (15.0) | | (9.2, 23.5) | | 12 (12.1) | | (7.0, 20.2) |
| **2 months** | 16 (16.0) | | (10.0, 24.6) | | 10 (10.0) | | (5.4, 17.7) | | 7 (7.3) | | (3.5, 14.6) | | 13 (13.0) | | (7.7, 21.2) | | 6 (6.1) | | (2.7, 12.9) |
| **3 months** | 24 (24.0) | | (16.6, 33.4) | | 24 (24.0) | | (16.6, 33.4) | | 35 (36.5) | | (27.4, 46.6) | | 35 (35.0) | | (26.2, 44.9) | | 28 (28.3) | | (20.2, 38.0) |
| **6 months** | 54 (54.0) | | (44.1, 63.6) | | 53 (53.0) | | (43.2, 62.6) | | 49 (51.0) | | (41.1, 60.9) | | 37 (37.0) | | (28.1, 46.9) | | 53 (53.5) | | (43.6, 63.2) |
|  |  | |  | |  | |  | |  | |  | |  | |  | |  | |  |
|  |  | | | | | | | | | | | | | | | | | | |
|  | **Providers** | | | | | | | | | | | | | | | | | | |
|  | **TRC  (N=20)**  **n (%)** | | **95% CI** | | **QSV  (N=10)**  **n (%)** | | **95% CI** | | **NKP  (N=9)**  **n (%)** | | **95% CI** | | **SRP  (N=11)**  **n (%)** | | **95% CI** | | **HY  (N=2)**  **n (%)** | | **95% CI** |
| **ART refill locations (more than one answer possible)** |  | |  | |  | |  | |  | |  | |  | |  | |  | |  |
| **ART clinics in hospitals** | 17 (85.0) | | (60.9, 95.4) | | 5 (50.0) | | (20.8, 79.2) | | 6 (66.7) | | (30.7, 90.0) | | 10 (90.9) | | (52.4, 98.9) | | 2 (100) | | - |
| **Other clinics in hospitals** | 5 (25.0) | | (10.3, 49.1) | | 4 (40.0) | | (14.5, 72.3) | | 6 (66.7) | | (30.7, 90.0) | | 0 | | - | | 0 | | - |
| **CBOs** | 8 (40.0) | | (20.7, 63.1) | | 8 (80.0) | | (42.9, 95.5) | | 5 (55.6) | | (23.1, 83.9) | | 4 (36.4) | | (13.2, 68.1) | | 1 (50.0) | | (1.8, 98.2) |
| **Primary care centers** | 15 (75.0) | | (50.9, 89.7) | | 8 (80.0) | | (42.9, 95.5) | | 8 (88.9) | | (45.5, 98.7) | | 4 (36.4) | | (13.2, 68.1) | | 2 (100) | | - |
| **Others** | 0 | | - | | 1 (10.0) | | (1.2, 50.8) | | 0 | | - | | 1 (9.1) | | (1.1, 47.6) | | 0 | | - |
| **ART refill providers (more than one answer possible)** |  | |  | |  | |  | |  | |  | |  | |  | |  | |  |
| **Physicians** | 16 (80.0) | | (55.8, 92.7) | | 9 (90.0) | | (49.2, 98.8) | | 9 (100) | | - | | 7 (63.6) | | (31.9, 86.8) | | 1 (50.0) | | (1.8, 98.2) |
| **Nurses** | 11 (55.0) | | (32.6, 75.5) | | 9 (90.0) | | (49.2, 98.8) | | 5 (55.6) | | (23.1, 83.9) | | 8 (72.7) | | (39.1, 91.7) | | 1 (50.0) | | (1.8, 98.2) |
| **Community health workers** | 5 (25.0) | | (10.3, 49.1) | | 3 (30.0) | | (9.0, 64.9) | | 1 (11.1) | | (1.3, 54.5) | | 3 (27.3) | | (8.3, 60.9) | | 0 | | - |
| **Health volunteers** | 1 (5.0) | | (0.6, 30.3) | | 2 (20.0) | | (4.5, 57.1) | | 1 (11.1) | | (1.3, 54.5) | | 1 (9.1) | | (1.1, 47.6) | | 0 | | - |
| **ART refill frequency** |  | |  | |  | |  | |  | |  | |  | |  | |  | |  |
| **Monthly** | 3 (15.0) | | (4.6, 39.1) | | 0 | | - | | 0 | | - | | 1 (9.1) | | (1.1, 47.6) | | 1 (50.0) | | (1.8, 98.2) |
| **2 months** | 0 | | - | | 0 | | - | | 0 | | - | | 1 (9.1) | | (1.1, 47.6) | | 0 | | - |
| **3 months** | 9 (45.0) | | (24.5, 67.4) | | 8 (80.0) | | (42.9, 95.5) | | 4 (44.4) | | (16.1, 76.9) | | 8 (72.7) | | (39.1, 91.7) | | 0 | | - |
| **6 months** | 8 (40.0) | | (20.7, 63.1) | | 2 (20.0) | | (4.5, 57.1) | | 5 (55.6) | | (23.1, 83.9) | | 1 (9.1) | | (1.1, 47.6) | | 1 (50.0) | | (1.8, 98.2) |
| **VL testing locations (more than one answer possible)** |  | |  | |  | |  | |  | |  | |  | |  | |  | |  |
| **ART clinics in hospitals** | 20 (100) | | - | | 6 (60.0) | | (27.7, 85.5) | | 6 (66.7) | | (30.7, 90.0) | | 10 (90.9) | | (52.4, 98.9) | | 2 (100) | | - |
| **Other clinics in hospitals** | 8 (40.0) | | (20.7, 63.1) | | 4 (40.0) | | (14.5, 72.3) | | 8 (88.9) | | (45.5, 98.7) | | 0 | | - | | 1 (50.0) | | (1.8, 98.2) |
| **CBOs** | 7 (35.0) | | (17.0, 58.6) | | 6 (60.0) | | (27.7, 85.5) | | 6 (66.7) | | (30.7, 90.0) | | 3 (27.3) | | (8.3, 60.9) | | 2 (100) | | - |
| **Primary care centers** | 9 (45.0) | | (24.5, 67.4) | | 8 (80.0) | | (42.9, 95.5) | | 9 (100) | | - | | 1 (9.1) | | (1.1, 47.6) | | 1 (50.0) | | (1.8, 98.2) |
| **Others** | 0 | | - | | 1 (10.0) | | (1.2, 50.8) | | 0 | | - | | 3 (27.3) | | (8.3, 60.9) | | 0 | | - |
| **VL testing providers (more than one answer possible)** |  | |  | |  | |  | |  | |  | |  | |  | |  | |  |
| **Physicians** | 20 (100) | | - | | 10 (100) | | - | | 9 (100) | | - | | 11 (100) | | - | | 2 (100) | | - |
| **Nurses** | 7 (35.0) | | (17.0, 58.6) | | 8 (80.0) | | (42.9, 95.5) | | 8 (88.9) | | (45.5, 98.7) | | 10 (90.9) | | (52.4, 98.9) | | 2 (100) | | - |
| **Community health workers** | 2 (10.0) | | (2.3, 34.0) | | 4 (40.0) | | (14.5, 72.3) | | 4 (44.4) | | (16.1, 76.9) | | 1 (9.1) | | (1.1, 47.6) | | 1 (50.0) | | (1.8, 98.2) |
| **Health volunteers** | 1 (5.0) | | (0.6, 30.3) | | 3 (30.0) | | (9.0, 64.9) | | 3 (33.3) | | (1.0, 69.3) | | 0 | | - | | 1 (50.0) | | (1.8, 98.2) |
| **VL testing frequency** |  | |  | |  | |  | |  | |  | |  | |  | |  | |  |
| **Monthly** | 0 | | - | | 0 | | - | | 0 | | - | | 0 | | - | | 1 (50.0) | | (1.8, 98.2) |
| **2 months** | 0 | | - | | 0 | | - | | 0 | | - | | 0 | | - | | 0 | | - |
| **3 months** | 4 (20.0) | | (7.3, 44.2) | | 2 (20.0) | | (4.5, 57.1) | | 3 (33.3) | | (10.0, 69.3) | | 0 | | - | | 0 | | - |
| **6 months** | 16 (80.0) | | (55.8, 92.7) | | 8 (80.0) | | (42.9, 95.5) | | 6 (66.7) | | (30.7, 90.0) | | 11 (100) | | - | | 1 (50.0) | | (1.8, 98.2) |
| **HIV/STI monitoring locations (more than one answer possible)** |  | |  | |  | |  | |  | |  | |  | |  | |  | |  |
| **ART clinics in hospitals** | 19 (95.0) | | (69.7, 99.4) | | 6 (60.0) | | (27.7, 85.5) | | 6 (66.7) | | (30.7, 90.0) | | 11 (100) | | - | | 2 (100) | | - |
| **Other clinics in hospitals** | 7 (35.0) | | (17.0, 58.6) | | 5 (50.0) | | (20.8, 79.2) | | 6 (66.7) | | (30.7, 90.0) | | 1 (9.1) | | (1.1, 47.6) | | 1 (50.0) | | (1.8, 98.2) |
| **CBOs** | 8 (40.0) | | (20.7, 63.1) | | 8 (80.0) | | (42.9, 95.5) | | 5 (55.6) | | (23.1, 83.9) | | 6 (54.5) | | (25.1, 81.1) | | 2 (100) | | - |
| **Primary care centers** | 12 (60.0) | | (36.9, 79.3) | | 8 (80.0) | | (42.9, 95.5) | | 9 (100) | | - | | 5 (45.5) | | (18.9, 74.9) | | 2 (100) | | - |
| **Others** | 0 | | - | | 1 (10.0) | | (1.2, 50.8) | | 0 | | - | | 1 (9.1) | | (1.1, 47.6) | | 0 | | - |
| **HIV/STI monitoring providers (more than one answer possible)** |  | |  | |  | |  | |  | |  | |  | |  | |  | |  |
| **Physicians** | 12 (60.0) | | (36.9, 79.3) | | 6 (60.0) | | (27.7, 85.5) | | 9 (100) | | - | | 9 (81.8) | | (46.5, 95.9) | | 1 (50.0) | | (1.8, 98.2) |
| **Nurses** | 15 (75.0) | | (50.9, 89.7) | | 6 (60.0) | | (27.7, 85.5) | | 8 (88.9) | | (45.5, 98.7) | | 10 (90.9) | | (52.4, 98.9) | | 2 (100) | | - |
| **Community health workers** | 10 (50.0) | | (28.5, 71.5) | | 8 (80.0) | | (42.9, 95.5) | | 7 (77.8) | | (38.8, 95.1) | | 8 (72.7) | | (39.1, 91.7) | | 2 (100) | | - |
| **Health volunteers** | 9 (45.0) | | (24.5, 67.4) | | 7 (70.0) | | (35.1, 91.0) | | 7 (77.8) | | (38.8, 95.1) | | 6 (54.5) | | (25.1, 81.1) | | 1 (50.0) | | (1.8, 98.2) |
| **HIV/STI monitoring frequency** |  | |  | |  | |  | |  | |  | |  | |  | |  | |  |
| **Monthly** | 3 (15.0) | | (4.6, 39.1) | | 0 | | - | | 2 (22.2) | | (4.9, 61.2) | | 2 (18.2) | | (4.1, 53.5) | | 1 (50.0) | | (1.8, 98.2) |
| **2 months** | 5 (25.0) | | (10.3, 49.1) | | 0 | | - | | 0 | | - | | 0 | | - | | 0 | | - |
| **3 months** | 6 (30.0) | | (13.6, 53.9) | | 8 (80.0) | | (42.9, 95.5) | | 4 (44.4) | | (16.1, 76.9) | | 5 (45.5) | | (18.9, 74.9) | | 1 (50.0) | | (1.8, 98.2) |
| **6 months** | 6 (30.0) | | (13.6, 53.9) | | 2 (20.0) | | (4.5, 57.1) | | 3 (33.3) | | (10.0, 69.3) | | 4 (36.4) | | (13.2, 68.1) | | 0 | | - |
| **Psychosocial support locations (more than one answer possible)** |  | |  | |  | |  | |  | |  | |  | |  | |  | |  |
| **ART clinics in hospitals** | 18 (90.0) | | (66.0, 97.7) | | 7 (70.0) | | (35.1, 91.0) | | 8 (88.9) | | (45.5, 98.7) | | 9 (81.8) | | (46.5, 95.9) | | 2 (100) | | - |
| **Other clinics in hospitals** | 6 (30.0) | | (13.6, 53.9) | | 4 (40.0) | | (14.5, 72.3) | | 8 (88.9) | | (45.5, 98.7) | | 2 (18.2) | | (4.1, 53.5) | | 1 (50.0) | | (1.8, 98.2) |
| **CBOs** | 16 (80.0) | | (55.8, 92.7) | | 8 (80.0) | | (42.9, 95.5) | | 7 (77.8) | | (38.8, 95.1) | | 9 (81.8) | | (46.5, 95.9) | | 2 (100) | | - |
| **Primary care centers** | 15 (75.0) | | (50.9, 89.7) | | 7 (70.0) | | (35.1, 91.0) | | 9 (100) | | - | | 7 (63.6) | | (31.9, 86.8) | | 2 (100) | | - |
| **Others** | 2 (10.0) | | (2.3, 34.0) | | 2 (2.0) | | (4.5, 57.1) | | 0 | | - | | 2 (18.2) | | (4.1, 53.5) | | 0 | | - |
| **Psychosocial support providers (more than one answer possible)** |  | |  | |  | |  | |  | |  | |  | |  | |  | |  |
| **Physicians** | 12 (60.0) | | (36.9, 79.3) | | 5 (50.0) | | (20.8, 79.2) | | 8 (88.9) | | (45.5, 98.7) | | 10 (90.9) | | (52.4, 98.9) | | 2 (100) | | - |
| **Nurses** | 12 (60.0) | | (36.9, 79.3) | | 6 (60.0) | | (27.7, 85.5) | | 7 (77.8) | | (38.8, 95.1) | | 11 (100) | | - | | 2 (100) | | - |
| **Community health workers** | 15 (75.0) | | (50.9, 89.7) | | 9 (90.0) | | (49.2, 98.8) | | 7 (77.8) | | (38.8, 95.1) | | 9 (81.8) | | (46.5, 95.9) | | 2 (100) | | - |
| **Health volunteers** | 17 (85.0) | | (60.9, 95.4) | | 9 (90.0) | | (49.2, 98.8) | | 7 (77.8) | | (38.8, 95.1) | | 9 (81.8) | | (46.5, 95.9) | | 2 (100) | | - |
| **Psychosocial support frequency** |  | |  | |  | |  | |  | |  | |  | |  | |  | |  |
| **Monthly** | 6 (30.0) | | (13.6, 53.9) | | 0 | | - | | 0 | | - | | 1 (9.1) | | (1.2, 47.6) | | 1 (50.0) | | (1.8, 98.2) |
| **2 months** | 4 (20.0) | | (7.3, 44.2) | | 1 (10.0) | | (1.2, 50.8) | | 2 (22.2) | | (4.9, 61.2) | | 2 (18.2) | | (4.1, 53.5) | | 0 | | - |
| **3 months** | 5 (25.0) | | (10.3, 49.1) | | 5 (50.0) | | (20.8, 79.2) | | 4 (44.4) | | (16.1, 76.9) | | 4 (36.4) | | (13.2, 68.1) | | 0 | | - |
| **6 months** | 5 (25.0) | | (10.3, 49.1) | | 4 (40.0) | | (14.5, 72.3) | | 3 (33.3) | | (10.0, 69.2) | | 4 (36.4) | | (13.2, 68.1) | | 1 (50.0) | | (1.8, 98.2) |

TRC, Thai Red Cross Anonymous Clinic; QSV, Queen Savang Vadhana Memorial Hospital; NKP, Nakornping Hospital; SRP, Sarapee Hospital; HY, Hatyai Hospital; 95% CI, 95% confidence interval; ART, antiretroviral therapy; CBOs, community-based organizations; VL, viral load; STI, sexually transmitted infection.
