# Supplementary material for: Client and provider preferences for HIV care: Implications for implementing differentiated service delivery in Thailand
Source: J Int AIDS Soc. 2021 Mar 31;24(4):e25693. doi: 10.1002/jia2.25693 (PMC8013790; doi:10.1002/jia2.25693)
Supplement: Supplementary file 7 — Appendix S1. Client and provider preferences for HIV care questionnaire: client version. Appendix S2. Client and provider preferences for HIV care questionnaire: provider version. [file JIA2-24-e25693-s006.docx]

**Appendix S1. Client and provider preferences for HIV care questionnaire: client version.**

| **Section 1:** Personal information | |
| --- | --- |
| 1. Date of birth: | _ _ /_ _ /_ _ _ _ |
| 1. Assigned sex at birth: | ❍ Male ❍ Female |
| 1. Identity that you want to express to others: | ❍ Male ❍ Female ❍ Gay man ❍ Kratoey ❍ Lesbian ❍ Transgender woman  ❍ Transgender man ❍ Other: _________ |
| 1. Education level: | ❍ Primary school (6^th^ grade and lower)  ❍ Secondary school (7^th^-12^th^ grade)/Vocational school/Higher vocational school  ❍ Bachelor’s degree  ❍ Master’s degree or higher  ❍ Other:_________ |
| **Section 2:** Service satisfaction | |
| 1. Are you satisfied with the health services at the ART clinic? | ❍ Yes ❍ No ❍ Not sure |
| 1. Are you satisfied with the providers at the ART clinic? | ❍ Yes ❍ No ❍ Not sure |
| 1. Are you confident in the ability of the providers at the ART clinic? | ❍ Yes ❍ No ❍ Not sure |
| 1. Are you satisfied with the manners and the attentiveness of the providers at the ART clinic? | ❍ Yes ❍ No ❍ Not sure |
| 1. Are you satisfied with the care and the assistance offered by the providers at the ART clinic? | ❍ Yes ❍ No ❍ Not sure |
| 1. Are you satisfied with the respect the providers express towards clients at the ART clinic? | ❍ Yes ❍ No ❍ Not sure |
| 1. Are you satisfied with the welcomeness of the providers at the ART clinic? | ❍ Yes ❍ No ❍ Not sure |
| 1. Are you satisfied with the sincerity of the providers at the ART clinic? | ❍ Yes ❍ No ❍ Not sure |
| 1. Are you satisfied with the privacy given by the providers at the ART clinic? | ❍ Yes ❍ No ❍ Not sure |
| 1. Are you satisfied with the appointment notification by the providers at the ART clinic? | ❍ Yes ❍ No ❍ Not sure |
| 1. Are you satisfied with the number of counselling rooms at the ART clinic? | ❍ Yes ❍ No ❍ Not sure |
| 1. Are you satisfied with the number of physicians at the ART clinic? | ❍ Yes ❍ No ❍ Not sure |
| 1. Are you satisfied with the operating hours of the ART clinic? | ❍ Yes ❍ No ❍ Not sure |
| 1. Are you satisfied with the service waiting time at the ART clinic? | ❍ Yes ❍ No ❍ Not sure |
| 1. Are you satisfied with the amount of time you meet with a physician at the ART clinic? | ❍ Yes ❍ No ❍ Not sure |
| 1. Is it convenient for you to travel to the ART clinic? | ❍ Yes ❍ No ❍ Not sure |
| 1. Are you satisfied with the overall medical service at the ART clinic? | ❍ Yes ❍ No ❍ Not sure |
| 1. Are you satisfied with the ART dispensing service at the ART clinic? | ❍ Yes ❍ No ❍ Not sure |
| 1. Are you satisfied with the CD4 count testing service at the ART clinic? | ❍ Yes ❍ No ❍ Not sure |
| 1. Are you satisfied with the risk reduction counselling at the ART clinic? | ❍ Yes ❍ No ❍ Not sure |
| 1. Are you satisfied with the adherence counselling at the ART clinic? | ❍ Yes ❍ No ❍ Not sure |
| 1. Are you satisfied with the STI screening service at the ART clinic? | ❍ Yes ❍ No ❍ Not sure |
| **Section 3:** Preferences for HIV care | |
| 1. Preferences for HIV care location (Multiple answers) | |
| - 1. Where would you like to start ART? | ❐ ART clinic in the hospital  ❐ Other clinic in the hospital  ❐ Community-based organization  ❐ Primary care centres  ❐ Other: _________ |
| - 1. Where would you like to refill ART in the long term? | ❐ ART clinic in the hospital  ❐ Other clinic in the hospital  ❐ Community-based organization  ❐ Primary care centres  ❐ Other: _________ |
| - 1. Where would you like to receive adherence counselling? | ❐ ART clinic in the hospital  ❐ Other clinic in the hospital  ❐ Community-based organization  ❐ Primary care centres  ❐ Other: _________ |
| - 1. Where would you like to test CD4 count? | ❐ ART clinic in the hospital  ❐ Other clinic in the hospital  ❐ Community-based organization  ❐ Primary care centres  ❐ Other: _________ |
| - 1. Where would you like to test viral load? | ❐ ART clinic in the hospital  ❐ Other clinic in the hospital  ❐ Community-based organization  ❐ Primary care centres  ❐ Other: _________ |
| - 1. Where would you like to screen for sexually transmitted infections? | ❐ ART clinic in the hospital  ❐ Other clinic in the hospital  ❐ Community-based organization  ❐ Primary care centres  ❐ Other: _________ |
| - 1. Where would you like to receive sexually transmitted infection treatment? | ❐ ART clinic in the hospital  ❐ Other clinic in the hospital  ❐ Community-based organization  ❐ Primary care centres  ❐ Other: _________ |
| - 1. Where would you like to receive HIV/STI monitoring? | ❐ ART clinic in the hospital  ❐ Other clinic in the hospital  ❐ Community-based organization  ❐ Primary care centres  ❐ Other: _________ |
| - 1. Where would you like to receive psychosocial support? | ❐ ART clinic in the hospital  ❐ Other clinic in the hospital  ❐ Community-based organization  ❐ Primary care centres  ❐ Other: _________ |
| - 1. Where would you like to receive risk reduction counselling? | ❐ ART clinic in the hospital  ❐ Other clinic in the hospital  ❐ Community-based organization  ❐ Primary care centres  ❐ Other: _________ |
| 1. Preferences for HIV care frequency | |
| - 1. How often would you like to refill ART? | ❍ Every month ❍ Every 2 months  ❍ Every 3 months ❍ Every 6 months |
| - 1. How often would you like to receive adherence counselling? | ❍ Every month ❍ Every 2 months  ❍ Every 3 months ❍ Every 6 months |
| - 1. How often would you like to receive CD4 count testing? | ❍ Every month ❍ Every 2 months  ❍ Every 3 months ❍ Every 6 months |
| - 1. How often would you like to receive viral load testing? | ❍ Every month ❍ Every 2 months  ❍ Every 3 months ❍ Every 6 months |
| - 1. How often would you like to screen for sexually transmitted infections? | ❍ Every month ❍ Every 2 months  ❍ Every 3 months ❍ Every 6 months |
| - 1. How often would you like to receive HIV/sexually transmitted infection monitoring? | ❍ Every month ❍ Every 2 months  ❍ Every 3 months ❍ Every 6 months |
| - 1. How often would you like to receive psychosocial support? | ❍ Every month ❍ Every 2 months  ❍ Every 3 months ❍ Every 6 months |
| - 1. How often would you like to receive risk reduction counselling? | ❍ Every month ❍ Every 2 months  ❍ Every 3 months ❍ Every 6 months |
| 1. Preferences for HIV care provider (Multiple answers) | |
| - 1. Who would you like to start ART? | ❐ Physicians  ❐ Nurses  ❐ Community health workers  ❐ Health volunteers |
| - 1. Who would you like to distribute ART? | ❐ Physicians  ❐ Nurses  ❐ Community health workers  ❐ Health volunteers |
| - 1. Who would you like to provide adherence counselling? | ❐ Physicians  ❐ Nurses  ❐ Community health workers  ❐ Health volunteers |
| - 1. Who would you like to order CD4 count testing? | ❐ Physicians  ❐ Nurses  ❐ Community health workers  ❐ Health volunteers |
| - 1. Who would you like to order viral load testing? | ❐ Physicians  ❐ Nurses  ❐ Community health workers  ❐ Health volunteers |
| - 1. Who would you like to order sexually transmitted infection screening tests? | ❐ Physicians  ❐ Nurses  ❐ Community health workers  ❐ Health volunteers |
| - 1. Who would you like to treat sexually transmitted infections? | ❐ Physicians  ❐ Nurses  ❐ Community health workers  ❐ Health volunteers |
| - 1. Who would you like to monitoring HIV/sexuality transmitted infections? | ❐ Physicians  ❐ Nurses  ❐ Community health workers  ❐ Health volunteers |
| - 1. Who would you like to provide psychosocial support? | ❐ Physicians  ❐ Nurses  ❐ Community health workers  ❐ Health volunteers |
| - 1. Who would you like to provide risk reduction counselling? | ❐ Physicians  ❐ Nurses  ❐ Community health workers  ❐ Health volunteers |
| **Section 4:** Expectation and concerns for differentiated service delivery | |
| 1. Differentiated service delivery would result in enhancement of retention in care: | ❍ Agree ❍ Disagree ❍ Not sure |
| 1. Differentiated service delivery would encourage your autonomy of a patient: | ❍ Agree ❍ Disagree ❍ Not sure |
| 1. Differentiated service delivery would empower your responsibility for health: | ❍ Agree ❍ Disagree ❍ Not sure |
| 1. Differentiated service delivery would strengthen the social network among clients: | ❍ Agree ❍ Disagree ❍ Not sure |
| 1. Differentiated service delivery provided by community health workers would result in less time waiting to refill ART: | ❍ Agree ❍ Disagree ❍ Not sure |
| 1. ART refill at community-based organizations would result in less time traveling to refill ART: | ❍ Agree ❍ Disagree ❍ Not sure |
| 1. ART refill at community-based organizations would reduce travel expense: | ❍ Agree ❍ Disagree ❍ Not sure |
| 1. ART refill at community-based organizations would reduce stigma: | ❍ Agree ❍ Disagree ❍ Not sure |
| 1. ART refill at community-based organizations would reduce discrimination: | ❍ Agree ❍ Disagree ❍ Not sure |
| 1. ART refill at community-based organizations would increase access to treatment: | ❍ Agree ❍ Disagree ❍ Not sure |
| 1. ART refill at community-based organizations would improve your attitude towards healthcare: | ❍ Agree ❍ Disagree ❍ Not sure |
| 1. Differentiated service delivery would result in poor adherence: | ❍ Agree ❍ Disagree ❍ Not sure |
| 1. Differentiated service delivery would increase loss to follow-up rate: | ❍ Agree ❍ Disagree ❍ Not sure |
| 1. Differentiated service delivery would a delayed detection of treatment failure: | ❍ Agree ❍ Disagree ❍ Not sure |
| 1. How the concerns for differentiated service delivery, such as loss to follow-up, poor adherence, and delayed detection of treatment failure, could be resolved? (Rank the most important 1-3 according to your opinion) | __ Clients should follow the ART clinic’s visit appointment  __ Health volunteers should continually assist in retaining and providing support to clients  __ Health volunteers should continually provide one-on-one counselling for clients  __ Community health workers should communicate with clients via online group message  __ Community health workers should communicate with clients via online direct message  __ Clients and health volunteers should communicate via web forums  __ Clients and community health workers should communicate via web forums  __ Clients should contact physicians or nurses directly via online platform  __ Other: _________ |

**Appendix S2. Client and provider preferences for HIV care questionnaire: provider version.**

| **Section 1:** Personal information | |
| --- | --- |
| 1. Date of birth: | _ _ /_ _ /_ _ _ _ |
| 1. Assigned sex at birth: | ❍ Male ❍ Female |
| 1. Identity that you want to express to others: | ❍ Male ❍ Female ❍ Gay man ❍ Kratoey ❍ Lesbian ❍ Transgender woman  ❍ Transgender man ❍ Other: _________ |
| 1. Workplace: | ❍ Hospital  ❍ Community-based organization  ❍ Provincial Health Office  ❍ Department of Disease Control  ❍ Other: _________ |
| 1. Position: | ❍ Physician  ❍ Nurse  ❍ Pharmacist  ❍ Medical technologist  ❍ Community health workers  ❍ Other: _________ |
| 1. Education level: | ❍ Primary school (6^th^ grade and lower)  ❍ Secondary school (7^th^-12^th^ grade)/Vocational school/Higher vocational school  ❍ Bachelor’s degree  ❍ Master’s degree or higher  ❍ Other: _________ |
| **Section 2:** Attitudes towards differentiated service delivery | |
| 1. Have you heard about differentiated service delivery? | ❍ Yes ❍ No ❍ Not sure |
| 1. Do you know the purposes of differentiated service delivery? | ❍ Yes ❍ No ❍ Not sure |
| 1. Do you know the benefits of differentiated service delivery? | ❍ Yes ❍ No ❍ Not sure |
| 1. Do you know the need for differentiated service delivery? | ❍ Yes ❍ No ❍ Not sure |
| 1. Do you agree with differentiated service delivery? | ❍ Yes ❍ No ❍ Not sure |
| 1. Do you think differentiated service delivery is appropriate for your setting? | ❍ Yes ❍ No ❍ Not sure |
| 1. Do you think differentiated service delivery is needed in your setting? | ❍ Yes ❍ No ❍ Not sure |
| **Section 3:** Preferences for HIV care | |
| 1. Preferences for HIV care location (Multiple answers) | |
| - 1. Where would you like ART initiation to take place? | ❐ ART clinic in the hospital  ❐ Other clinic in the hospital  ❐ Community-based organization  ❐ Primary care centres  ❐ Other: _________ |
| - 1. Where would you like long-term ART refill to be provided? | ❐ ART clinic in the hospital  ❐ Other clinic in the hospital  ❐ Community-based organization  ❐ Primary care centres  ❐ Other: _________ |
| - 1. Where would you like adherence counselling to be provided? | ❐ ART clinic in the hospital  ❐ Other clinic in the hospital  ❐ Community-based organization  ❐ Primary care centres  ❐ Other: _________ |
| - 1. Where would you like CD4 count testing to be provided? | ❐ ART clinic in the hospital  ❐ Other clinic in the hospital  ❐ Community-based organization  ❐ Primary care centres  ❐ Other: _________ |
| - 1. Where would you like viral load testing to be provided? | ❐ ART clinic in the hospital  ❐ Other clinic in the hospital  ❐ Community-based organization  ❐ Primary care centres  ❐ Other: _________ |
| - 1. Where would you like sexually transmitted infection screening to be provided? | ❐ ART clinic in the hospital  ❐ Other clinic in the hospital  ❐ Community-based organization  ❐ Primary care centres  ❐ Other: _________ |
| - 1. Where would you like sexually transmitted infection treatment to be provided? | ❐ ART clinic in the hospital  ❐ Other clinic in the hospital  ❐ Community-based organization  ❐ Primary care centres  ❐ Other: _________ |
| - 1. Where would you like HIV/STI monitoring to be provided? | ❐ ART clinic in the hospital  ❐ Other clinic in the hospital  ❐ Community-based organization  ❐ Primary care centres  ❐ Other: _________ |
| - 1. Where would you like psychosocial support to be provided? | ❐ ART clinic in the hospital  ❐ Other clinic in the hospital  ❐ Community-based organization  ❐ Primary care centres  ❐ Other: _________ |
| - 1. Where would you like to risk reduction counselling to be provided? | ❐ ART clinic in the hospital  ❐ Other clinic in the hospital  ❐ Community-based organization  ❐ Primary care centres  ❐ Other: _________ |
| 1. Preferences for HIV care frequency | |
| - 1. How often would you like refill ART to be provided? | ❍ Every month ❍ Every 2 months  ❍ Every 3 months ❍ Every 6 months |
| - 1. How often would you like adherence counselling to be provided? | ❍ Every month ❍ Every 2 months  ❍ Every 3 months ❍ Every 6 months |
| - 1. How often would you like CD4 count testing to be provided? | ❍ Every month ❍ Every 2 months  ❍ Every 3 months ❍ Every 6 months |
| - 1. How often would you like viral load testing to be provided? | ❍ Every month ❍ Every 2 months  ❍ Every 3 months ❍ Every 6 months |
| - 1. How often would you like sexually transmitted infection screening to be provided? | ❍ Every month ❍ Every 2 months  ❍ Every 3 months ❍ Every 6 months |
| - 1. How often would you like HIV/sexually transmitted infection monitoring to be provided? | ❍ Every month ❍ Every 2 months  ❍ Every 3 months ❍ Every 6 months |
| - 1. How often would you like psychosocial support to be provided? | ❍ Every month ❍ Every 2 months  ❍ Every 3 months ❍ Every 6 months |
| - 1. How often would you like risk reduction counselling to be provided? | ❍ Every month ❍ Every 2 months  ❍ Every 3 months ❍ Every 6 months |
| 1. Preferences for HIV care provider (Multiple answers) | |
| - 1. Who would you like to start ART? | ❐ Physicians  ❐ Nurses  ❐ Community health workers  ❐ Health volunteers |
| - 1. Who would you like to distribute ART? | ❐ Physicians  ❐ Nurses  ❐ Community health workers  ❐ Health volunteers |
| - 1. Who would you like to provide adherence counselling? | ❐ Physicians  ❐ Nurses  ❐ Community health workers  ❐ Health volunteers |
| - 1. Who would you like to order CD4 count testing? | ❐ Physicians  ❐ Nurses  ❐ Community health workers  ❐ Health volunteers |
| - 1. Who would you like to order viral load testing? | ❐ Physicians  ❐ Nurses  ❐ Community health workers  ❐ Health volunteers |
| - 1. Who would you like to order sexually transmitted infection screening tests? | ❐ Physicians  ❐ Nurses  ❐ Community health workers  ❐ Health volunteers |
| - 1. Who would you like to treat sexually transmitted infections? | ❐ Physicians  ❐ Nurses  ❐ Community health workers  ❐ Health volunteers |
| - 1. Who would you like to monitoring HIV/sexuality transmitted infections? | ❐ Physicians  ❐ Nurses  ❐ Community health workers  ❐ Health volunteers |
| - 1. Who would you like to provide psychosocial support? | ❐ Physicians  ❐ Nurses  ❐ Community health workers  ❐ Health volunteers |
| - 1. Who would you like to provide risk reduction counselling? | ❐ Physicians  ❐ Nurses  ❐ Community health workers  ❐ Health volunteers |
| **Section 4:** Expectation and concerns for differentiated service delivery | |
| 1. Differentiated service delivery would result in enhancement of retention in care: | ❍ Agree ❍ Disagree ❍ Not sure |
| 1. Differentiated service delivery would encourage client autonomy of a patient: | ❍ Agree ❍ Disagree ❍ Not sure |
| 1. Differentiated service delivery would empower client responsibility for health: | ❍ Agree ❍ Disagree ❍ Not sure |
| 1. Differentiated service delivery would strengthen the social network among clients: | ❍ Agree ❍ Disagree ❍ Not sure |
| 1. Differentiated service delivery provided by community health workers would result in less time waiting to refill ART: | ❍ Agree ❍ Disagree ❍ Not sure |
| 1. ART refill at community-based organizations would result in less time traveling to refill ART: | ❍ Agree ❍ Disagree ❍ Not sure |
| 1. ART refill at community-based organizations would reduce travel expense: | ❍ Agree ❍ Disagree ❍ Not sure |
| 1. ART refill at community-based organizations would reduce stigma: | ❍ Agree ❍ Disagree ❍ Not sure |
| 1. ART refill at community-based organizations would reduce discrimination: | ❍ Agree ❍ Disagree ❍ Not sure |
| 1. ART refill at community-based organizations would increase access to treatment: | ❍ Agree ❍ Disagree ❍ Not sure |
| 1. ART refill at community-based organizations would improve client attitude towards healthcare: | ❍ Agree ❍ Disagree ❍ Not sure |
| 1. Differentiated service delivery would reduce the workload of hospital providers: | ❍ Agree ❍ Disagree ❍ Not sure |
| 1. Differentiated service delivery would result in poor adherence: | ❍ Agree ❍ Disagree ❍ Not sure |
| 1. Differentiated service delivery would increase loss to follow-up rate: | ❍ Agree ❍ Disagree ❍ Not sure |
| 1. Differentiated service delivery would a delayed detection of treatment failure: | ❍ Agree ❍ Disagree ❍ Not sure |
| 1. Differentiated service delivery would increase the workload at community-based organizations: | ❍ Agree ❍ Disagree ❍ Not sure |
| 1. Regulatory restrictions would be a barrier for differentiated service delivery: | ❍ Agree ❍ Disagree ❍ Not sure |
| 1. How the concerns for differentiated service delivery, such as loss to follow-up, poor adherence and delayed detection of treatment failure, could be resolved? (Rank the most important 1-3 according to your opinion) | __ Clients should follow the ART clinic’s visit appointment  __ Health volunteers should continually assist in retaining and providing support to clients  __ Health volunteers should continually provide one-on-one counselling for clients  __ Community health workers should communicate with clients via online group message  __ Community health workers should communicate with clients via online direct message  __ Clients and health volunteers should communicate via web forums  __ Clients and community health workers should communicate via web forums  __ Clients should contact physicians or nurses directly via online platform |
